# A novel hybrid model integrating CEEMDAN decomposition, dispersion entropy and LSTM for photovoltaic power forecasting and anomaly detection

**DOI:** 10.1038/s41598-025-23305-3

**Published:** 2025-11-11

**Authors:** Ziqi Qiu, Jiarong Ye, Jiahui Lu, Nenghui Zhu

**Affiliations:** 1 School of Opto-electronic and Communication Engineering, Xiamen University of Technology, Xiamen, 361024 China; 2 School of Mathematics and Statistics, Xiamen University of Technology, Xiamen, 361024 China

**Keywords:** PV power forecasting, CEEMDAN, Dispersion entropy, LSTM neural network, Hybrid model, Anomaly detection, Energy science and technology, Engineering, Mathematics and computing

## Abstract

Photovoltaic (PV) power generation exhibits significant non-stationary characteristics due to the influence of meteorological conditions and equipment status, which makes traditional prediction methods difficult to accurately capture its dynamic variations and abnormal behaviors. To address these limitations, a CEEMDAN-DispEn-LSTM hybrid framework is proposed for PV power forecasting and anomaly detection. Following preprocessing via the Median Absolute Deviation (MAD) method and decomposition using the Complete Ensemble Empirical Mode Decomposition with Adaptive Noise (CEEMDAN), optimal components are selected in this study through a dual-criterion approach that concurrently accounts for energy proportion and correlation coefficient. Dispersion Entropy (DispEn) is employed to quantify signal complexity, while dedicated Long Short-Term Memory (LSTM) subnetworks integrated with entropy weighting are utilized to dynamically achieve multi-scale feature fusion. Furthermore, dual deviation logic is adopted to detect non-meteorological anomalies. Experimental results confirm that the proposed framework outperforms selected benchmark models across most prediction metrics. In anomaly detection, the framework demonstrates significant effectiveness in identifying line faults and PID effects, while exhibiting preliminary capability in detecting partial shading. The latter finding points to a clear direction for future performance enhancement through multi-source data fusion. Thus, this study establishes a validated technical pathway for non-stationary time series forecasting, particularly suited for ultra-short-term power prediction and anomaly detection in distributed photovoltaic systems under temperate climates, highlighting its application potential in the operation and maintenance of such systems.

## Introduction

Against the backdrop of accelerating global energy transition^1^, photovoltaic (PV) power generation has become increasingly pivotal in the energy supply system. However, its output exhibits complex time-varying and non-stationary characteristics due to meteorological factors and equipment status^2^, posing challenges to grid stability and energy management. Traditional methods fail to accurately capture dynamic variations or equipment anomalies under complex conditions.

Research in PV forecasting and anomaly detection has advanced via hardware sensing and big data approaches. Hardware-based methods enable component-level monitoring^3^ but incur high costs, while big data methods leverage historical-meteorological correlations for superior adaptability^4^. Prediction models have evolved from single to hybrid systems: LSTM serves as a temporal modeling benchmark^5,6^; CNN-LSTM fuses spatio-temporal features^7,8^; GRU-CNN optimizes short-term prediction^9^; and multi-channel LSTM/MT-RNN enhances multi-plant/user generalization^10–12^. Transformers excel in long-term dependency modeling^13^ but are computationally expensive, whereas MHA improves feature interaction^14,15^.

However, the strong non-stationarity of PV power signals hinders single prediction models from fully extracting effective features. Thus, signal decomposition technology has become key to improving prediction accuracy, with its development as follows: EEMD suffers from mode aliasing^16–18^; NA-MEMD struggles to separate noise^19^; VMD is parameter-sensitive^20^; while CEEMDAN achieves high-quality decomposition via adaptive noise^21^. Entropy algorithms have also advanced: ApEn suits short-sequence analysis^22^; SampEn reduces length sensitivity^18^; FuzzyEn enhances stability^17^; PermEn ignores amplitude differences^22,23^; and DispEn, with amplitude-sensitive mapping, becomes the most robust amplitude-frequency feature indicator^23^. Notably, the CEEMDAN-PE-BiLSTM framework integrates “decomposition-entropy” innovatively^21^, providing a technical reference but failing to solve modal screening and multi-scale modeling issues. Additionally, anomaly detection relies on hardware diagnosis^3^ or prediction residual analysis^4^, with this study adopting the latter big data-driven approach.

Although the aforementioned technologies lay a foundation for PV forecasting, existing methods still exhibit three core limitations in practice: (1) CEEMDAN modal reconstruction uses fixed entropy thresholds, ignoring energy-correlation synergy^20^; (2) Models struggle with time-scale differences (RNN gradient vanishing^6^, CNNs’ inadequate long-term capture^9^, Transformers’ overfitting^13,16^; (3) Hybrid architectures lack complexity-driven dynamic weight allocation for high/low-frequency interaction^8,14–16^.

This study focuses on small-to-medium distributed PV systems and edge real-time scenarios—characterized by small capacity, limited data, restricted edge computing resources, and high sensitivity to local meteorology (e.g., building shading, local clouds). Despite cloud image/meteorological field methods’ superiority in medium-long-term forecasting, time-series data is preferred for: (1) Accessibility (inverter-sensor collection^4^; (2) Ultra-short-term (5–30 min) adaptability^6^; (3) Anomaly detection compatibility via residual analysis^3^.

Supplementary comparisons with state-of-the-art methods support the selection of LSTM. Specifically, Transformer-based models (including Informer^24,25^ achieve strong accuracy at daily scales, they typically require extensive training data and suffer from marked performance degradation on smaller datasets. Moreover, these architectures demand substantially greater computational resources compared to the more efficient LSTM framework, limiting their practicality in real-time scenarios. Transformer-based models are also prone to overfitting high-frequency noise, whereas LSTM’s gating mechanism offers more robust adaptation to multi-scale temporal features^6^. Though stacked models^26,27^ enhance generalization, they introduce parameter redundancy and higher deployment costs.

In view of this, this paper proposes the CEEMDAN-DispEn-LSTM model under a “decomposition-screening-fusion” framework, with three improvements:

- Multi-dimensional feature screening: CEEMDAN decomposes signals, and energy-correlation criteria filter valid IMFs, which reduces the interference of redundant components compared to single entropy threshold screening;
- Dynamic fusion mechanism: Independent LSTM sub-networks with complexity-based weights model fluctuations and trends, enabling more targeted feature learning for components with different complexity;
- Cross-scale collaborative modeling: LSTM gating adapts to diurnal cycles and sudden weather changes, improving the model’s ability to capture temporal features of different scales.

This framework is expected to improve the accuracy of PV forecasting and the reliability of anomaly detection compared to existing models, providing a reference for the optimization of non-stationary time series prediction methods in PV systems. The remainder of this paper is structured as follows: “Methodology” introduces research methods (CEEMDAN, DispEn, LSTM, and residual-based anomaly detection); “Data preprocessing” elaborates on data preprocessing using the SOLETE dataset; “Model construction” constructs the CEEMDAN-DispEn-LSTM framework; “Results and analysis” compares prediction and anomaly detection performance via the SOLETE dataset and a Chinese distributed PV measured dataset; “Conclusions” summarizes advantages, discusses limitations, and prospects future research.

## Methodology

**Fig. 1:**
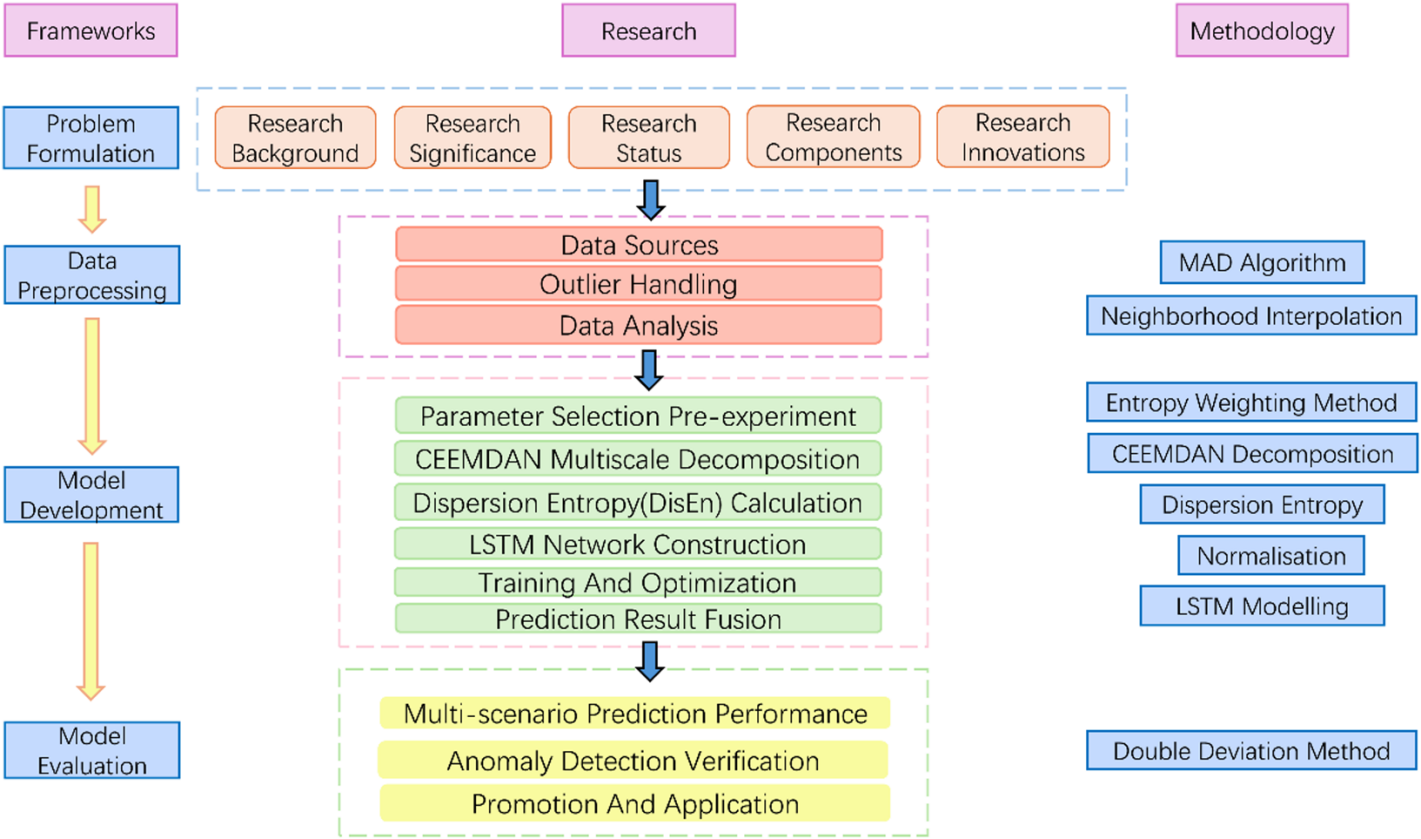
Research methodology flowchart.

First, the collected PV power generation data is preprocessed: The MAD method is employed to detect outliers, followed by imputation using the neighborhood interpolation method. During model construction, pre-experiments are conducted to screen parameters using the entropy weight method. Subsequently, CEEMDAN is applied to decompose the data, and DispEn is calculated to quantify signal complexity. Based on this, an LSTM neural network is constructed for dynamic fusion modeling, and the fused prediction results are generated after training and optimization. Finally, the model performance is evaluated across multiple scenarios using the test set, and the double-deviation method is applied to validate the anomaly detection capability, thereby assessing the model’s effectiveness and engineering applicability. Figure 1 illustrates the flowchart of the research methodology.

### CEEMDAN decomposition

CEEMDAN^28^ is a signal processing method improved from EEMD^29^. EEMD solves the mode mixing problem existing in Empirical Mode Decomposition (EMD) by adding white noise, but it has drawbacks such as residual noise. CEEMDAN further optimizes the noise processing and mode decomposition processes, effectively improving the accuracy of signal decomposition and noise reduction capability. The core steps of CEEMDAN are as follows:

Step 1. Initialization: define the original signal as $\:x\left(t\right)$.

Step 2. Generate a set of noisy signals $\:{x}_{i}\left(t\right)\:(\mathrm{i}=1,\:2,\:3,\:\:...,\:\mathrm{N})$:

1 $$\:\begin{array}{c}{x}_{i}\left(t\right)=x\left(t\right)+\epsilon\:*{\omega\:}_{i}\left(t\right),\end{array}$$

where $\:{\omega\:}_{i}\left(t\right)$ is the white noise. The noise intensity $\:\epsilon\:$ is dynamically adjusted according to the decomposition level. Adding white noise to the original signal can enhance its multi-scale characteristic differences, facilitating subsequent decomposition.

Step 3. Break down the IMF step by step.

First-order IMF ($\:IM{F}_{1}$): Perform EMD decomposition on each noisy signal $\:{x}_{i}\left(t\right)$ to extract the first mode $\:IM{F}_{1}^{\left(i\right)}\left(t\right)$, and take the meaning of all $\:IM{F}_{1}^{\left(i\right)}\left(t\right)$ as the final $\:IM{F}_{1}$:

2 $$\:\begin{array}{c}IM{F}_{1}\left(t\right)=\frac{1}{N}\sum\:_{i=1}^{N}IM{F}_{1}^{\left(i\right)}\left(t\right),\end{array}$$

Update residuals:

3 $$\:\begin{array}{c}{r}_{1}\left(t\right)=x\left(t\right)-IM{F}_{1}\left(t\right).\end{array}$$

Subsequent hierarchical decomposition ($\:k\ge\:\:2$): Repeat steps 2–3 for the residual $\:{r}_{k-1}\left(t\right)$ to generate the $\:k$-th layer IMF:

4 $$\:\begin{array}{c}IM{F}_{k}\left(t\right)=\frac{1}{N}\sum\:_{i=1}^{N}IMD\left({r}_{k-1}\left(t\right)+{\epsilon\:}_{k}*{\omega\:}_{i}\left(t\right)\right),\end{array}$$

where $\:{\epsilon\:}_{k}$ is adjusted according to hierarchical adaptation (usually with gradual attenuation).

Update residuals:

5 $$\:\begin{array}{c}{r}_{k}\left(t\right)={r}_{k-1}\left(t\right)-IM{F}_{k}\left(t\right),\end{array}$$

Terminate the decomposition when the residual $\:{r}_{k}\left(t\right)\:$becomes a monotonic function or has insufficient extreme points.

Step 4. Output results:

6 $$\:\begin{array}{c}x\left(t\right)=\sum\:_{k=1}^{K}IM{F}_{k}\left(t\right)+{r}_{k}\left(t\right).\end{array}$$

### Long short-term memory

**Fig. 2:**
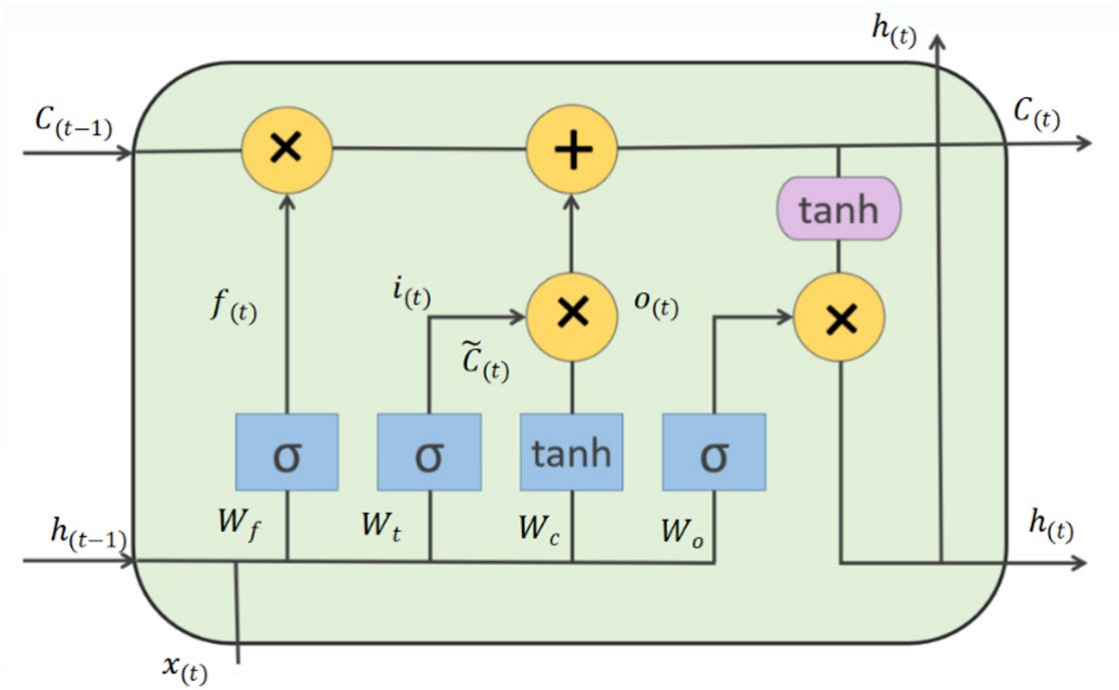
LSTM principal diagram for long-term and short-term memory networks.

As a classic deep learning model for time series prediction, LSTM is widely applied in PV power forecasting. LSTM effectively mitigates the gradient vanishing/exploding issues inherent in traditional RNN via a gating mechanism and excels at capturing long-term dependencies in time series. This study adopts the LSTM architecture proposed by Graves and Schmidhuber^30^, which is illustrated in Fig. 2.

Assume that the time step is $\:t$, the input is $\:{x}_{t}$ the hidden state is $\:{h}_{t-1}$, the cell state is $\:{C}_{t-1}$, the weight matrix is $\:W$, the bias is $\:b$, and the activation functions are $\:Sigmoid\left(\sigma\:\right)$and $\:\mathrm{T}\mathrm{a}\mathrm{n}\mathrm{h}$.

The forgetting gate is used to calculate forgetting probability, and its output $\:{f}_{t}$ is:

7 $$\:\begin{array}{c}{f}_{t}=\sigma\:\left({W}_{f}*\left[{h}_{t-1},{x}_{t}\right]+{b}_{f}\right).\end{array}$$

The input gate is used to calculate the input probability and new candidate values. The output $\:{i}_{t}$ of the forget gate and the candidate cell state $\:\stackrel{\sim}{{C}_{t}}$ are:

8 $$\:\begin{array}{c}{i}_{t}=\sigma\:\left({W}_{i}*\left[{h}_{t-1},{x}_{t}\right]+{b}_{i}\right),\end{array}$$

9 $$\:\begin{array}{c}\stackrel{\sim}{{C}_{t}}=\mathrm{tanh}\left({W}_{c}*\left[{h}_{t-1},{x}_{t}\right]+{b}_{c}\right).\end{array}$$

By combining the results of the forgetting gate and the input gate, the cell state can be updated, and the output gate controls the memory unit $\:{C}_{t}$:

10 $$\:\begin{array}{c}{C}_{t}={f}_{t}\odot {C}_{t-1}+{i}_{t} \odot \stackrel{\sim}{{C}_{t}},\end{array}$$

Discard useless information through $\:{f}_{t}$ ,then add new information through $\:{i}_{t}$and $\:\stackrel{\sim}{{C}_{t}}$. ⨀ usually denotes element-wise multiplication (Hadamard product).

The output gate is used to calculate the output probability and generate the current hidden state:

11 $$\:\begin{array}{c}{o}_{t}=\sigma\:\left({W}_{0}*\left[{h}_{t-1},{x}_{t}\right]+{b}_{o}\right),\end{array}$$

12 $$\:\begin{array}{c}{h}_{t}={o}_{t} \odot tanh{(C}_{t}).\end{array}$$

Based on the updated cell state $\:{C}_{t}$, determine the output $\:{h}_{t}$ for the current time step.

### Dispersion entropy

Dispersion entropy^31^ (DispEn) is a metric for measuring the irregularity of time series, and it plays a significant role in fields such as signal processing. DispEn focuses on describing the complexity and irregularity of signals. The calculation steps are as follows:

First, subsequence partitioning: Extract subsequences of length m in order from the given time series. For example, if the time series is $\:\left\{{x}_{1},{x}_{2},\dots\:,{x}_{n}\right\}$ and m = 3, then the sub-sequences may be $\:[{x}_{1},{x}_{2},{x}_{3}]$, $\:[{x}_{2},{x}_{3},{x}_{4}]$, etc.

Second, numerical sorting and difference calculation: Sort the values in each subsequence in ascending order, then calculate the differences between adjacent values. For example, if the subsequence is [3,1,2], after sorting it becomes [1,2,3], and the differences between adjacent numbers are 2 − 1 = 1 and 3 − 2 = 1.

Next, determine the probability distribution: Observe the frequency distribution of differences. For example, in the above example, the difference of 1 appeared twice. If only this single subsequence is considered, the probability of the difference being 1 is 1.

Finally, calculating the entropy value:

13 $$\:\begin{array}{c}H\left(X\right)=-\sum\:\left(p\left(x\right)\mathrm{log}\left(p\left(x\right)\right)\right),\end{array}$$

where $\:H\left(X\right)$ is the DispEn, and $\:p\left(x\right)$is the probability density function of the data value x. The higher the entropy value, the more complex and irregular the signal.

### Double deviation detection logic

A double deviation method is proposed to determine the threshold, identifying abnormal situations in PV power generation operation and maintenance. First, define the following deviation indicators:

Absolute deviation (ΔP): Calculates the absolute difference between the actual power and the predicted power at each moment (Used to detect large power changes):

14 $$\:\begin{array}{c}\varDelta\:P=\left|{\mathrm{P}}_{\mathrm{a}\mathrm{c}\mathrm{t}\mathrm{u}\mathrm{a}\mathrm{l}}-{\mathrm{P}}_{\mathrm{p}\mathrm{r}\mathrm{e}\mathrm{d}\mathrm{i}\mathrm{c}\mathrm{t}\mathrm{e}\mathrm{d}}\right|.\end{array}$$

Relative deviation (ΔP/P): Calculates the ratio of the absolute deviation to the actual power when the actual power is not zero. (Used to detect relative changes, especially during periods of low power or near-zero power:

15 $$\:\begin{array}{c}\frac{{\Delta\:}\mathrm{P}}{\mathrm{P}}=\frac{{\Delta\:}\mathrm{P}}{{\mathrm{P}}_{\mathrm{a}\mathrm{c}\mathrm{t}\mathrm{u}\mathrm{a}\mathrm{l}}}.\end{array}$$

Based on the normal operation data of this model, calculate the mean (µ) and standard deviation (σ) of the absolute deviation and relative deviation, and set the dynamic threshold as follows:

16 $$\:\begin{array}{c}Upper\:threshold\:limit=\mu\:+3\sigma,\end{array}$$

17 $$\:\begin{array}{c}Lower\:threshold\:limit=\mu\:-3\sigma. \end{array}$$

When both the absolute deviation and relative deviation exceed the corresponding thresholds, it is marked as a non-weather-related abnormality. Through a dual verification mechanism, misjudgments based on a single indicator are avoided, improving the accuracy of anomaly detection.

### The entropy weighting method

The entropy weighting method is an objective weighting approach based on data variability, with its core foundation in information entropy theory^32^: the greater the data variability (the lower the entropy value), the higher the weight; conversely, the lower the weight. The following are the implementation steps.

Data standardization:

18 $$\:\begin{array}{c}Z={\left({z}_{ij}\right)}_{m\times\:n},\end{array}$$

Positive indicators (the higher the value, the better):

19 $$\:\begin{array}{c}{z}_{ij}=\frac{{x}_{ij}-{min}_{j}{x}_{j}}{{max}_{j}{x}_{j}-{min}_{j}{x}_{j}}; \end{array}$$

Negative indicators (the lower the value, the better):

20 $$\:\begin{array}{c}{z}_{ij}=\frac{{max}_{j}{x}_{j}-{x}_{ij}}{{max}_{j}{x}_{j}-{min}_{j}{x}_{j}}.\end{array}$$

Calculating entropy value:

21 $$\:\begin{array}{c}{E}_{j}=-\frac{1}{\mathrm{ln}m}\sum\:_{i=1}^{m}\left(\frac{{z}_{ij}}{\sum\:_{i=1}^{m}{z}_{ij}}\right)\mathrm{ln}\left(\frac{{z}_{ij}}{\sum\:_{i=1}^{m}{z}_{ij}}\right),\end{array}$$

The smaller $\:{E}_{j}$ is, the greater the degree of variation in the indicator.

Calculate the difference coefficient:

22 $$\:\begin{array}{c}{d}_{j}=1-{E}_{j}.\#\left(22\right)\end{array}$$

Determine the indicator’s weight:

23 $$\:\begin{array}{c}{w}_{j}=\frac{{d}_{j}}{\sum\:_{j=1}^{n}{d}_{j}},\end{array}$$

The final weight vector $\:w=({w}_{1},{w}_{2}\dots\:{w}_{n})$ objectively reflects the contribution of each indicator to the evaluation.

### Data preprocessing

#### Data sources

This study utilizes the SOLETE dataset^33^ from the Wind Energy and Energy Systems Laboratory (SYSLAB) at the Technical University of Denmark (DTU), with a sampling interval of 5 min. The data covers a monitoring area in Denmark where an 11-kW wind turbine and a 10-kW PV inverter are co-located. To focus on the objectives of this study, we exclusively use PV inverter data. This method eliminates interference from multiple data sources, focusing on analyzing the operational characteristics of the PV inverter and exploring the intrinsic relationships between power output and factors such as irradiance and temperature. This provides reliable data support for research on PV inverter performance optimization and early fault warning.

**Table 1:** Influencing factors and variables for PV power generation.

Impact factor	An explanation of the meaning of words or phrases
Temperature	Temperature in degrees Celsius (°C) corresponding to the point in time of the observation
Array plane irradiance	The radiant power per unit area irradiated into the plane of a PV array, usually in watts per square meter (W/m²)
Wind speed	Wind speed in meters per second (m/s)
Relative humidity	The ratio of the temperature at which the air is saturated with water vapor content (dew point) to the corresponding temperature at the point in time of the observation, usually expressed as a percentage
Actual power	Actual power output of the PV system at a given point in time, in kilowatts (kW)

As a clean energy technology converting sunlight to electricity, PV power generation is significantly affected by meteorological factors. Due to data acquisition limitations, this model primarily considers key meteorological influences on generation efficiency as listed in Table 1.

### Outlier handling and data preprocessing

PV power generation data is affected by factors such as light intensity and temperature, exhibiting complex characteristics including time-variability, periodicity, and abrupt change points. However, traditional methods fail to effectively adapt to these characteristics, leading to frequent misjudgment of outliers and reduced data reliability. To address this issue, this study adopts the MAD algorithm^34^ to handle data anomalies. Based on median calculation, this method has strong robustness against extreme values and outliers, which is specified as follows:

24 $$\:\begin{array}{c}{M}_{MAD}=b\times\:{median}_{i=1,\dots\:,n}\left(\left|{x}_{i}-{median}_{j=1,\dots\:,n}\left({x}_{j}\right)\right|\right),\end{array}$$

where b is a constant, usually taken as 1.4826.

To detect outliers in the observation data, it is necessary to further calculate the discrimination coefficient D for each observation value $\:{x}_{i}$:

25 $$\:\begin{array}{c}D=\frac{{x}_{i}-{median}_{j=1,\dots\:,n}\left({x}_{j}\right)}{{M}_{MAD}}, \end{array}$$

In this study, $\:{x}_{i}$ is classified as an outlier when $\:D>3$.

**Fig. 3:**
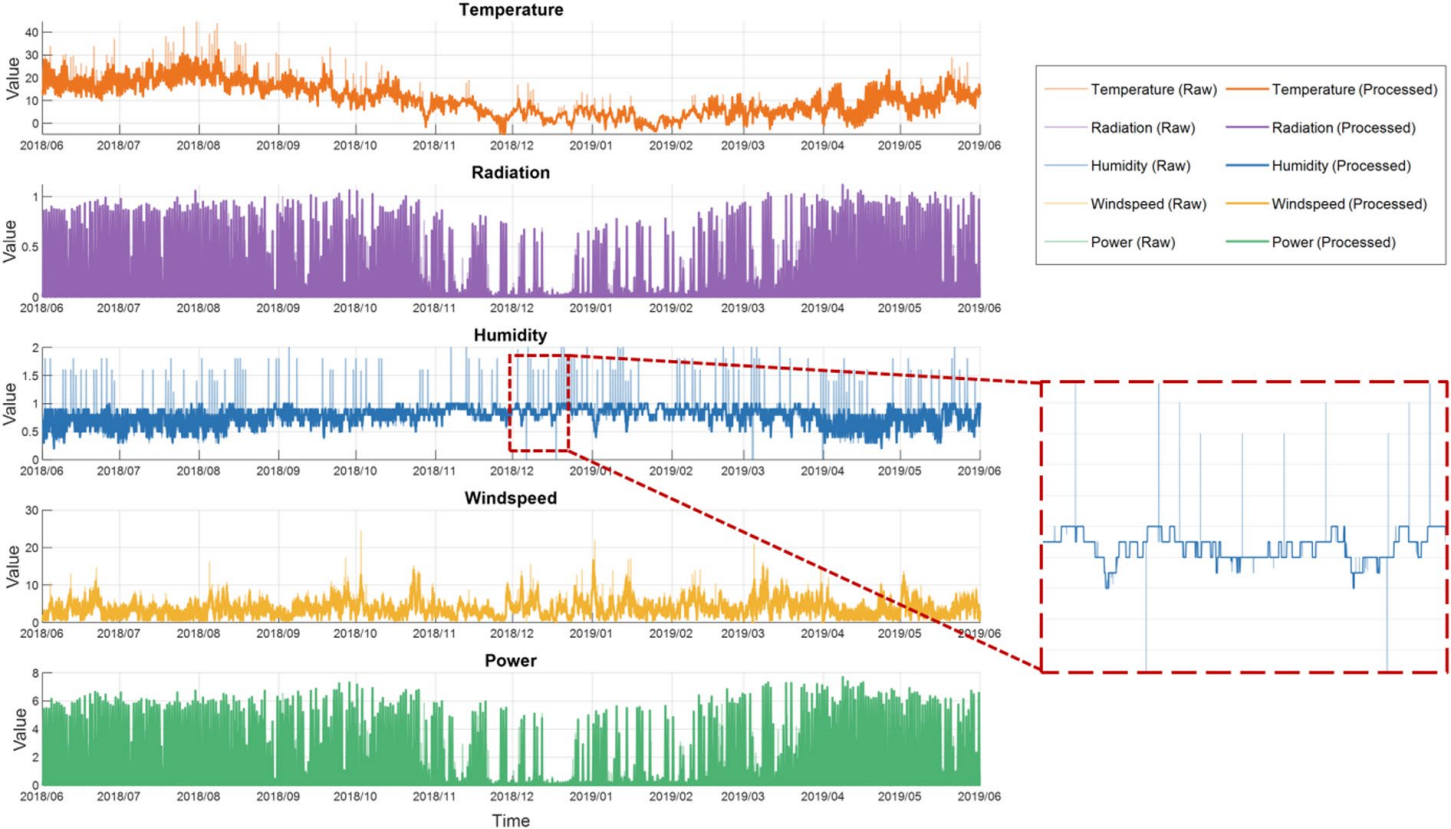
Meteorological factors and PV power: time-series before and after preprocessing.

After preprocessing with the MAD algorithm, this paper adopts the method of averaging preceding and subsequent data points for secondary processing of residual outliers to ensure the smoothness and stability of the data. To systematically analyze the annual operating characteristics of PV inverters, this study selects the complete annual data from June 2018 to June 2019 as the scope of analysis. The effects of data before and after preprocessing are shown in Fig. 3.

The dataset was divided: Data from June 1, 2018 to September 30, 2018 was used as the training set, which covers various weather conditions and enables sufficient learning of the operating characteristics of PV inverters; Data from October 1, 2018 to November 30, 2018 was selected as the test set, during which there were significant weather fluctuations while avoiding extreme weather in December. The testing and training set are temporally contiguous but non-overlapping, which can both verify the generalization ability of the model and effectively prevent overfitting.

**Fig. 4:**
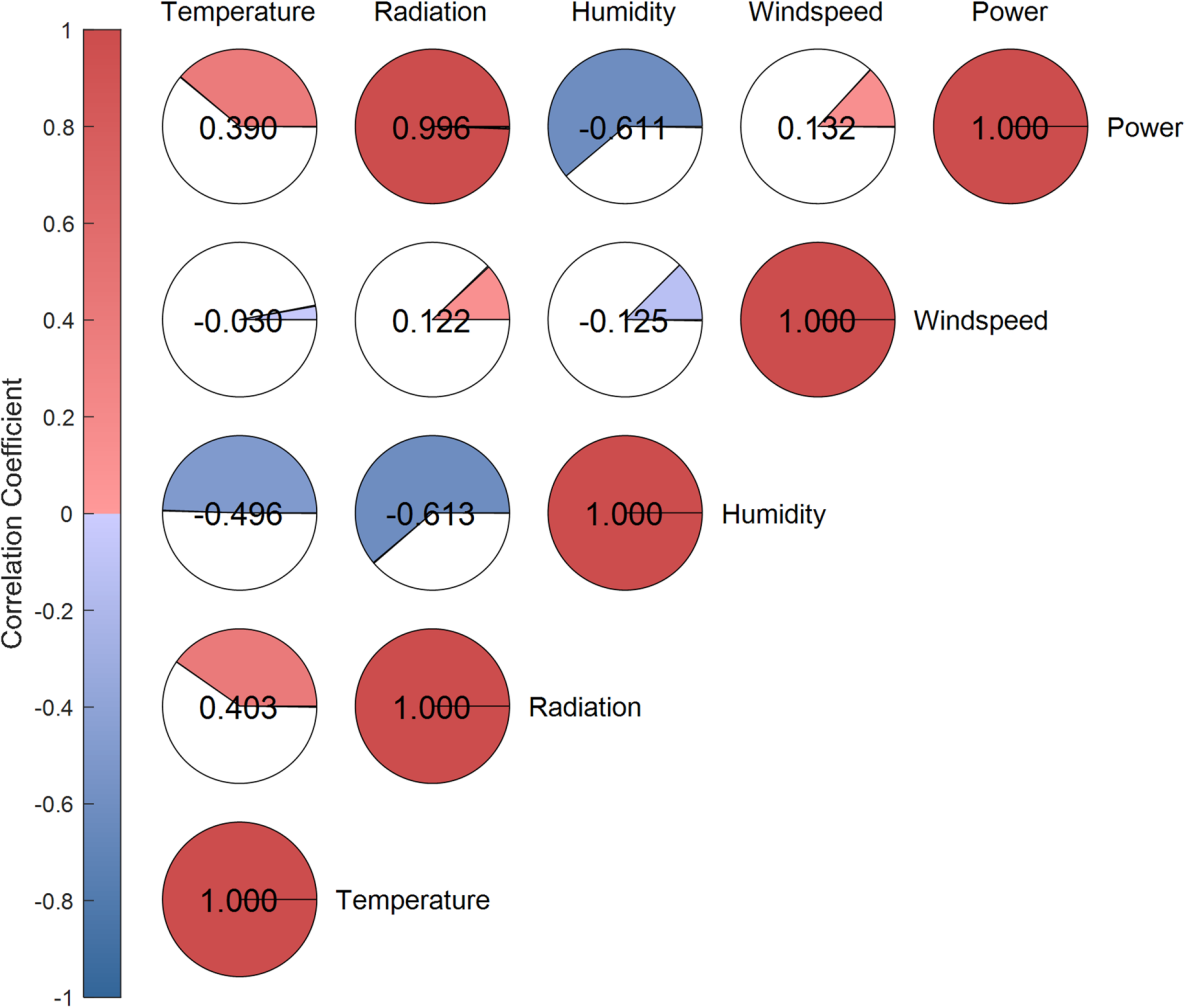
Visualization of correlation coefficients between variables.

**Fig. 5:**
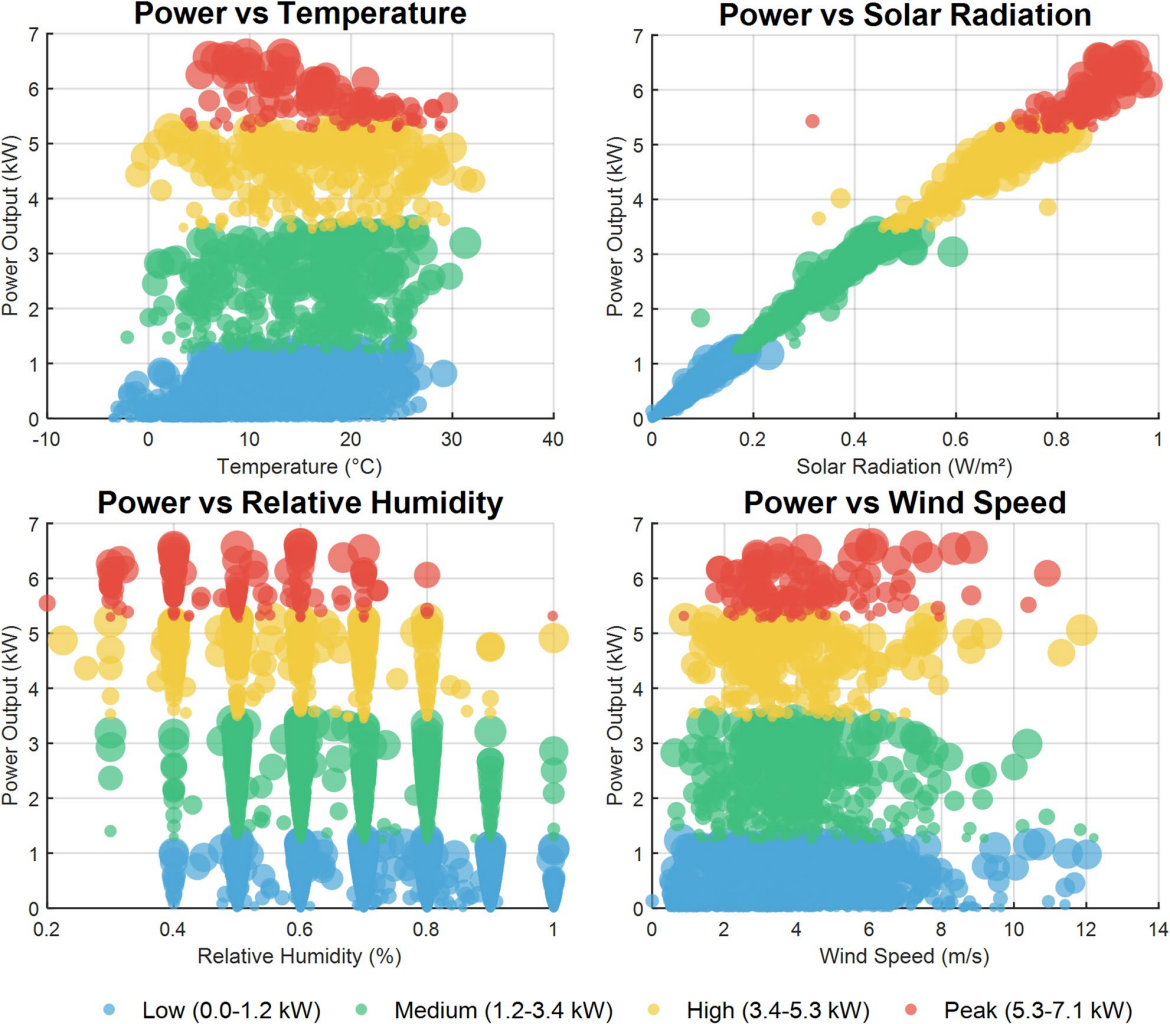
Bubble diagram of power versus other meteorological factors.

Factor division: As shown in Figs. 4 and 5, the power generation has a strong positive correlation with solar irradiance, a moderate positive correlation with ambient temperature, a moderate negative correlation with relative humidity, and appears to have no significant correlation with wind speed. Therefore, solar irradiance is identified as the core factor affecting PV power generation, with temperature and humidity as secondary factors.

In addition, to eliminate differences in feature dimensions, min-max normalization^35^ is adopted in data preprocessing. This method linearly maps data of all dimensions to the [0,1] interval, which not only ensures the stability of subsequent DispEn calculations but also significantly improves the learning efficiency of the LSTM network for multi-source time-series features.

### Model construction

**Fig. 6:**
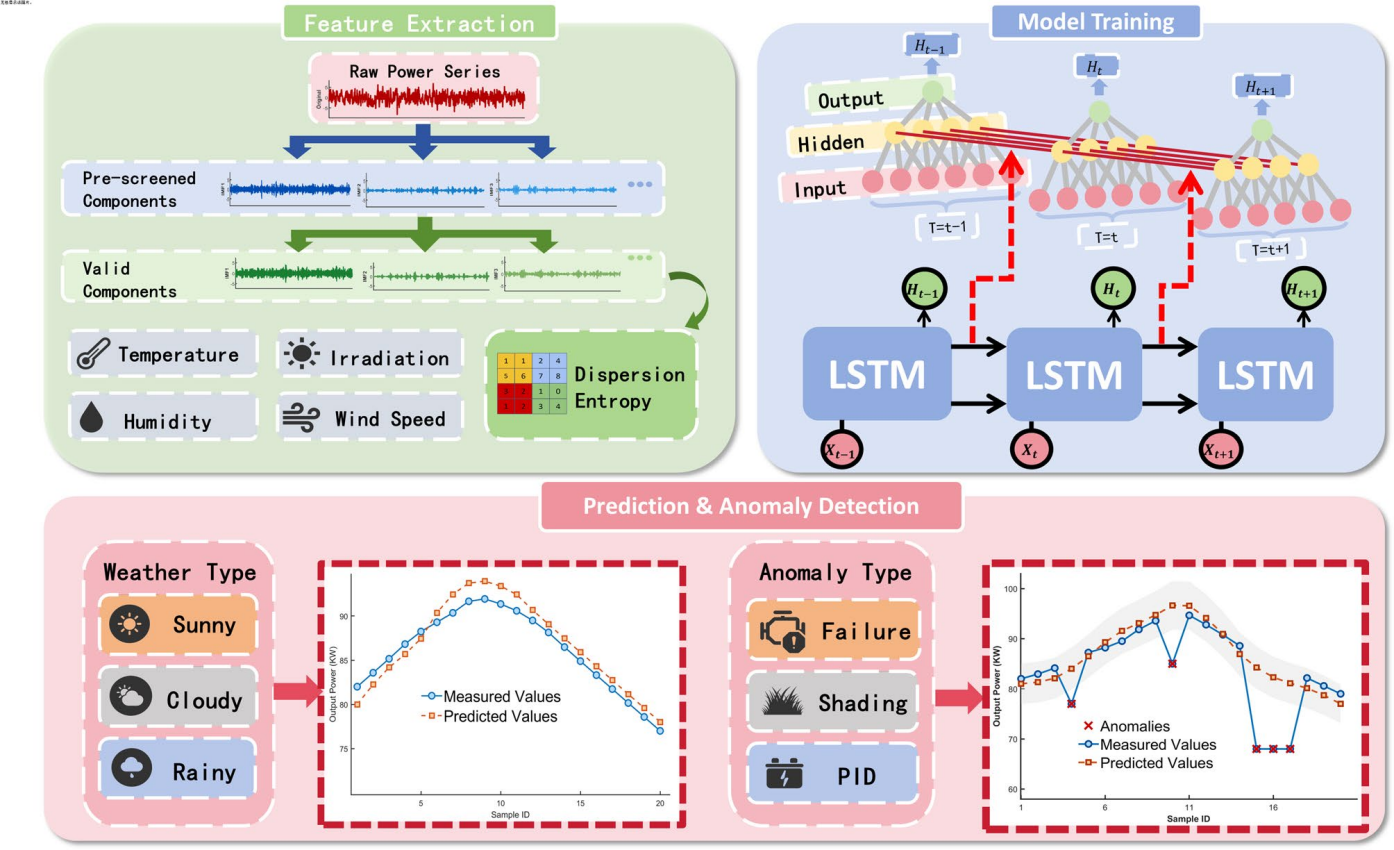
Main flowchart of model construction.

The model obtains 16 Intrinsic Mode Function (IMF) components through CEEMDAN decomposition. It then calculates the DispEn of each component and normalizes it to the [0,1] interval to provide standardized features for subsequent weighted synthesis. After inputting the standardized feature data into the weight-independent LSTM networks for training, the model synthesizes and outputs multiple prediction results through weighted summation. The flow chart of the model is shown in Fig. 6.

### Preliminary experiment on multi-model parameter combination selection

This study conducted preliminary experiments using the entropy weight method, scientifically quantifying indicator weights to determine the optimal parameters for the CEEMDAN, scatter entropy, and LSTM models.​

#### CEEMDAN model parameter preliminary experiment

CEEMDAN model involves two parameters: the noise intensity (Nstd) is set to {0.1, 0.2, 0.3}, and the number of noise realizations (NR) is set to 50, 100, 150, 200, forming 12 parameter combinations. For each combination, the entropy weight method is used to determine the weights of the spectral overlap degree ($\:SO$), the proportion of effective IMFs ($\:EIMF$), and the computation efficiency ($\:CE$). The comprehensive scoring formula:

26 $$\:\begin{array}{c}Score=\:0.296\times\:SO+\:0.135\times\:EIMF+\:0.569\times\:CE.\end{array}$$

**Fig. 7:**
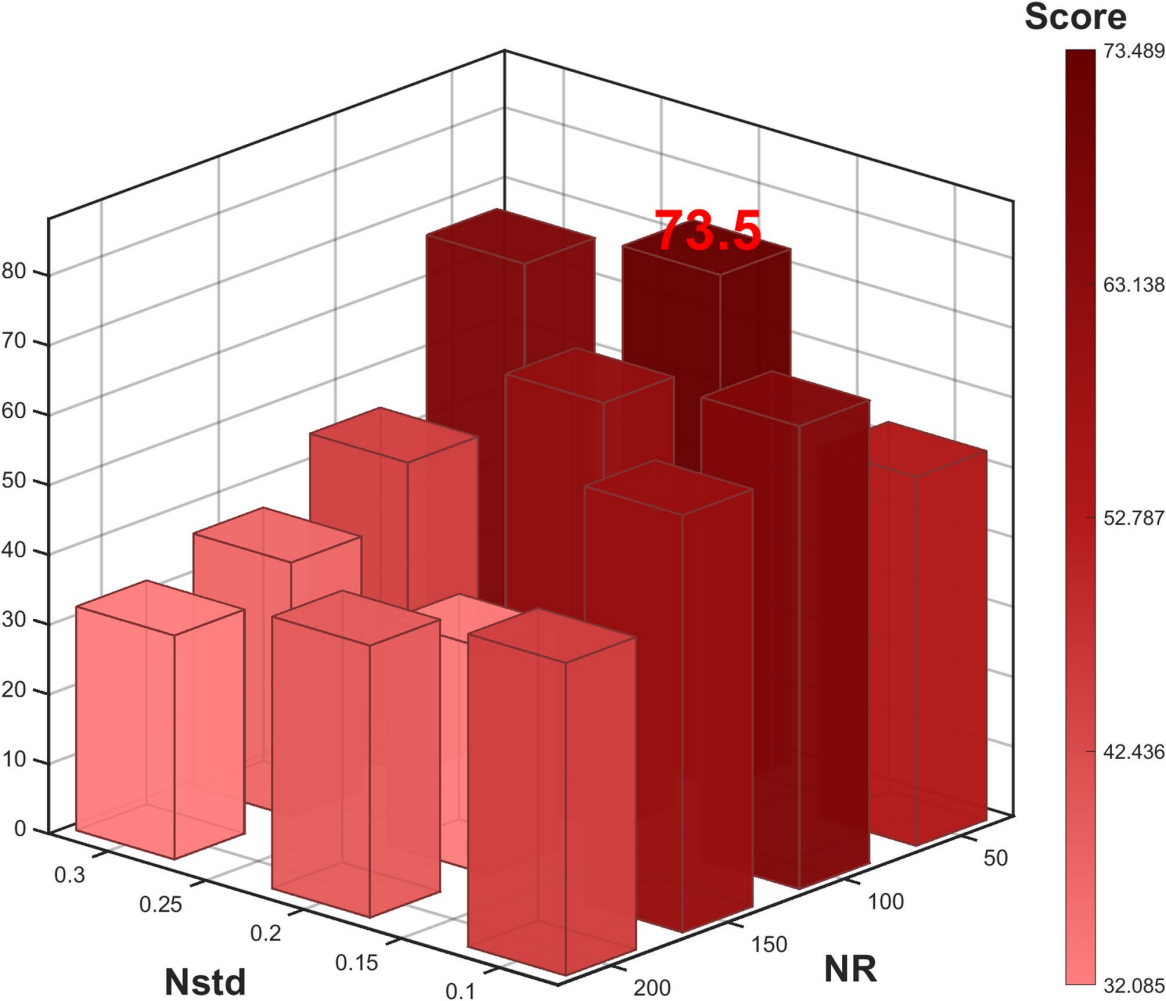
CEEMDAN performance 3D heat histogram based on $\:\boldsymbol{N}\boldsymbol{s}\boldsymbol{t}\boldsymbol{d}$ and $\:\boldsymbol{N}\boldsymbol{R}$ parameter.

Figure 7 shows the highest overall score (73.5) at Nstd = 0.2 and NR = 50, with spectral overlap of 0.0857, effective IMF ratio of 0.94, and processing time of 66.36 s, thus determining this as CEEMDAN’s optimal parameter set.

LSTM parameter preliminary experiment. The number of hidden layers (Depth) was set to {1, 2, 3} and the number of neurons (Neurons) was set to {16, 32, 64, 128}, forming 12 parameter combinations. After standardizing RMSE, MAE, R², and computation time ($\:CE$), their entropy weights were determined. The final score formula:

27 $$\:\begin{array}{c}Score=\:\:0.305\times\:RMSE\:+\:0.222\times\:MAE+0.277\times\:{R}^{2}+0.196\times\:CE.\end{array}$$

**Fig. 8:**
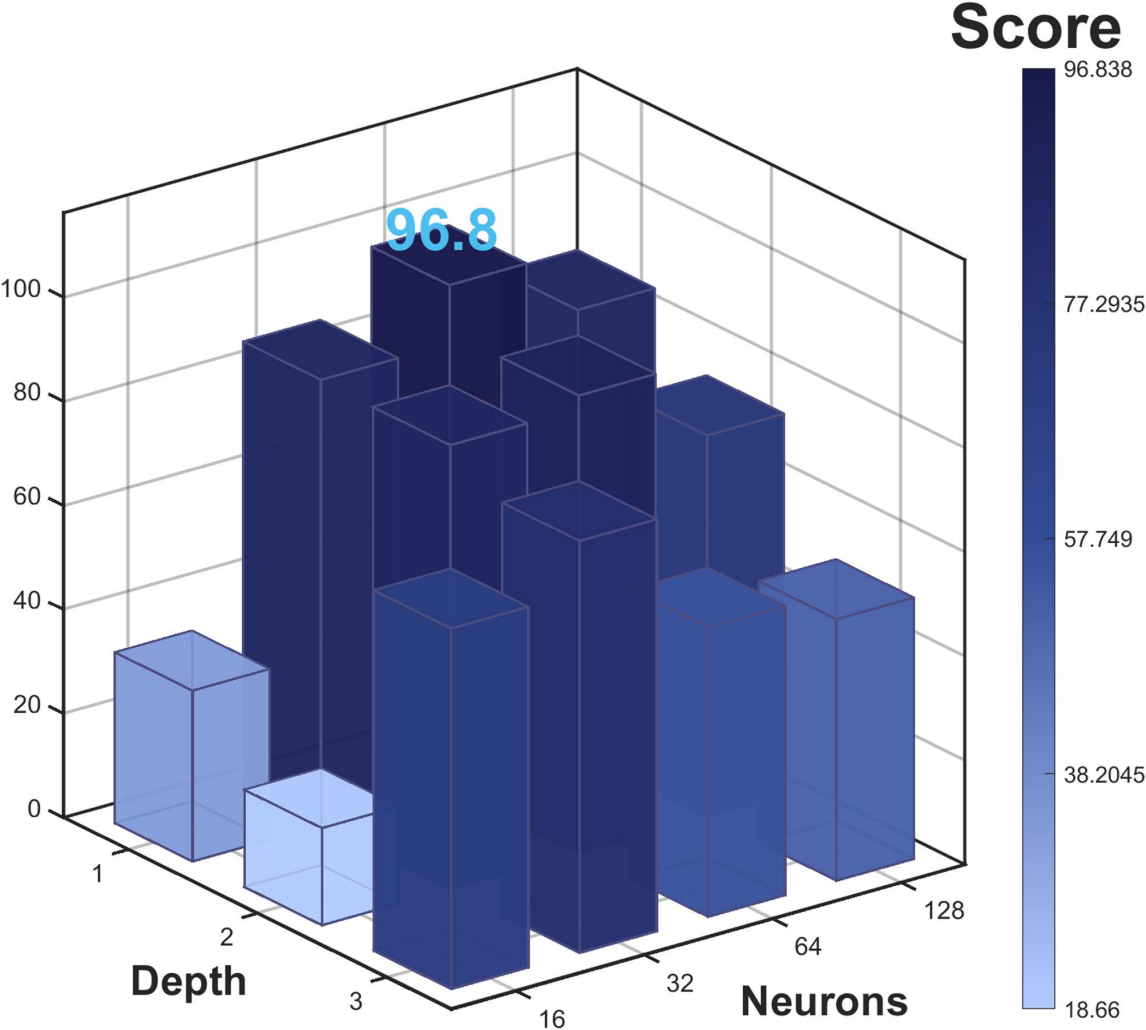
LSTM performance 3D heat histogram based on depth and neurons parameters.

As shown in Fig. 8, when Depth is 1 and Neurons is 64, the comprehensive score is highest (96.8), which is determined to be the optimal structure combination for LSTM.

#### Preliminary experiment on DispEn parameters

The DispEn quality metric and computation time were used as evaluation criteria. The embedding dimension (m) was set to {2, 3, 4}, the number of categories (c) to {3, 4, 5, 6}, and the time delay (τ) to {1, 2, 3} to generate parameter combinations. The score:

28 $$\:\begin{array}{c}Score=\:0.717\times\:Q\:+\:0.283\times\:CE. \end{array}$$

**Fig. 9:**
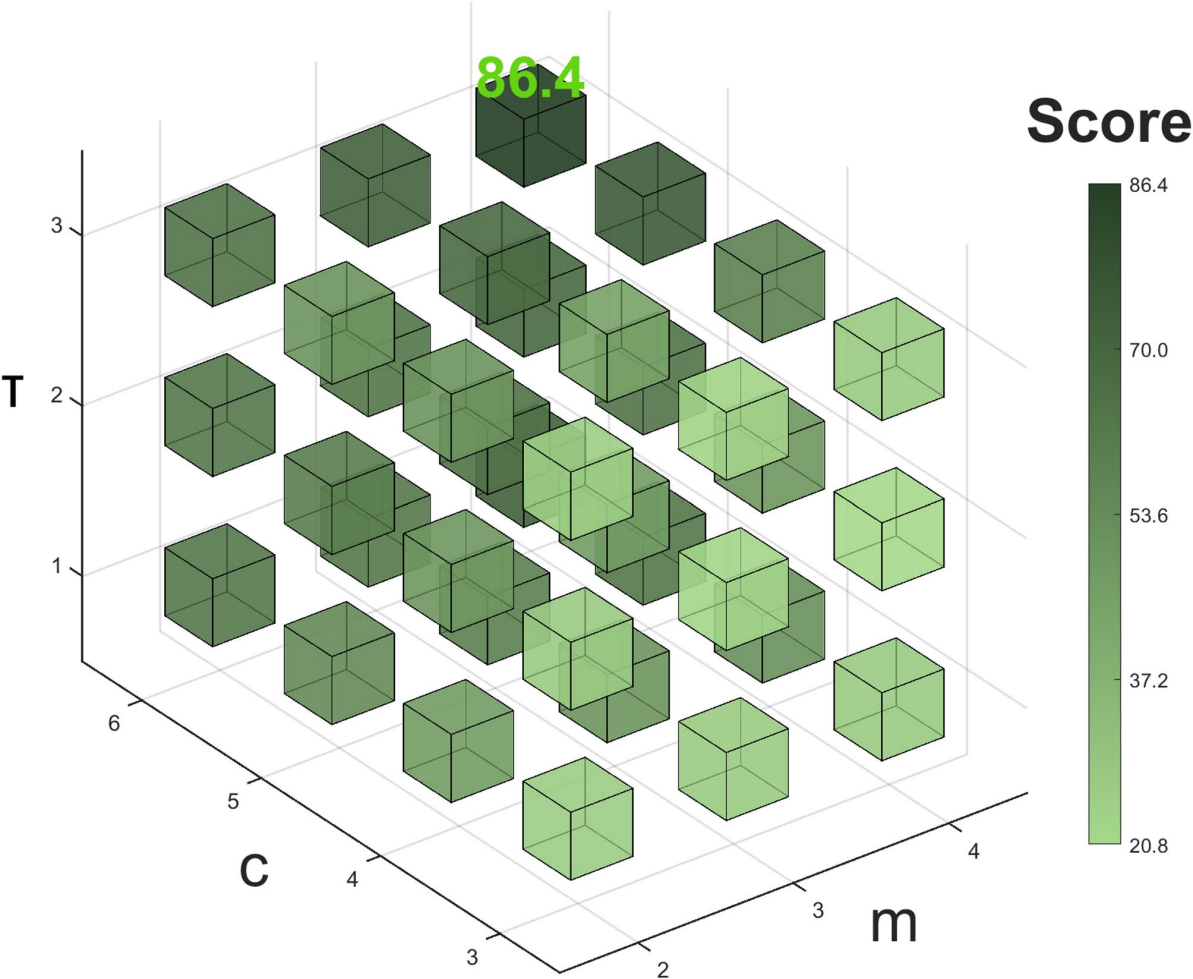
DispEn performance 3D Rubik’s heatmap based on $\:\boldsymbol{m}$, $\:\boldsymbol{c}$, and $\:\boldsymbol{\tau\:}$ parameters.

Figure 9 shows that when the embedding dimension $\:m\:=\:4$, the number of categories $\:c\:=\:6$, and the time delay $\:\tau\:\:=\:3$, the comprehensive score is the highest (86.4). Therefore, this set of parameters is determined as the optimal combination for DispEn.

### Modal decomposition and screening based on CEEMDAN

**Fig. 10:**
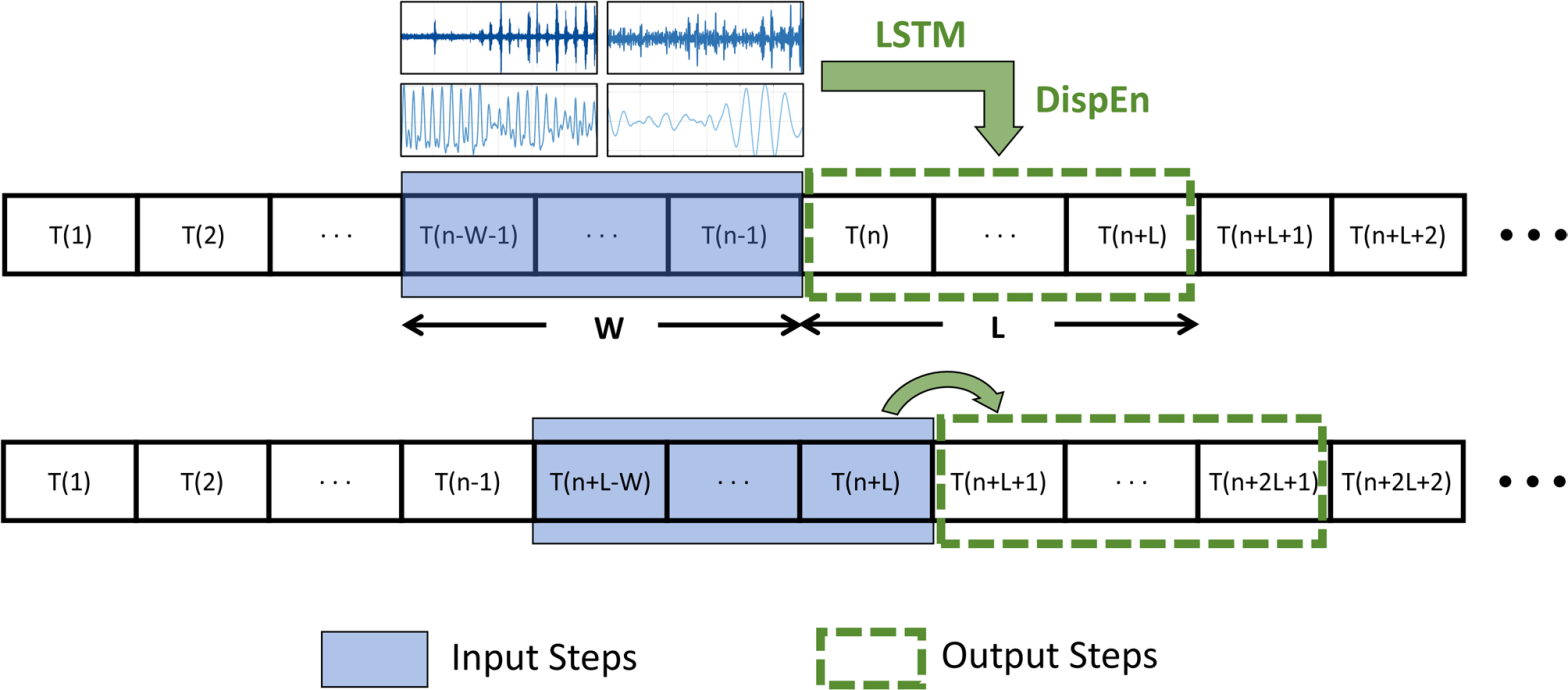
Illustration of the rolling-window based decomposition and prediction methodology.

To prevent data leakage, a rolling window CEEMDAN decomposition method^36^ is employed. Within each window of length W, the following operations are performed sequentially: First, the photovoltaic power sequence within the window undergoes CEEMDAN decomposition to yield multiple IMF components; then, redundant components are dynamically eliminated through dual screening (energy proportion < 0.5% or correlation coefficientρ < 0.1); The filtered IMF components are fed into an LSTM model for prediction; finally, the weights of each IMF component are calculated using dispersion entropy, and weighted synthesis is performed to obtain the final prediction output. The prediction stride is L. Figure 10 visually demonstrates the effectiveness of rolling decomposition and filtering, proving enhanced feature capture capability and prediction accuracy.

### Weight-independent LSTM modeling and training

Step 1 - Feature Fusion: Concatenate normalized climate features, current IMF component data, and normalized approximate entropy values to form a multi-source input feature vector.

Step 2 - Sample Construction: Use sliding window technology to combine feature vectors at consecutive time points with corresponding future power values into training samples.

Step 3 - Format Conversion: Convert sample data into the cell format required by the LSTM network.​.

Step 4 -Network Construction: Determine through preliminary experiments to adopt an LSTM structure with a single hidden layer (64 neurons).​.

 Step 5 - Training Configuration: Use the Adam optimizer^37^, set the maximum number of training epochs to 50, batch size to 64, learning rate decay strategy (10% decay every 5 epoch), and apply L2 regularization to suppress overfitting; train the model in a CPU environment.​

 Step 6 - Prediction and Denormalization: After predicting the training and test sets, convert the results to the original power scale through denormalization^38^, and save the prediction results of each component simultaneously.

### Calculate the dispen

For each IMF component obtained from the CEEMDAN decomposition, calculate its DispEn to quantify the dynamic complexity of each component. Subsequently, normalize the DispEn by mapping its range to $\:[0,\:1]$ to provide standardized features for subsequent weighted synthesis.

### Weighted synthesis of multiple prediction results

Using the normalized DispEn values of each IMF component as a basis, calculate the weighting coefficients and combine the prediction results of all components through weighted summation to highlight the contribution of complex features to the prediction. Then, apply Gaussian filtering^39^ to the combined results of the test set to smooth out short-term fluctuations and improve the stability of the prediction curve.

## Results and analysis

### Comparison of model performance under different weather conditions

#### Selected model

To validate the effectiveness of CEEMDAN-DispEn-LSTM, the following models were selected for comparison experiments:

- Reference model group: GRU, LSTM, Transformer^40^, Dlinear^41^.
- Improved model group: CEEMDAN-LSTM, CEEMDAN-DispEn-LSTM.

In the comparison models, GRU and LSTM serve as reference models: GRU has a simple structure and efficient training and can be regarded as a simplified version of LSTM; while LSTM, with its gating mechanism, excels at capturing long-term dependencies in time series. CEEMDAN-LSTM introduces modal decomposition technology into the base LSTM model; The CEEMDAN-DispEn-LSTM model further incorporates DispEn based on CEEMDAN-LSTM. Additionally, we include two mainstream time series forecasting models, Transformer and Dlinear, in the baseline models for comparison.

#### Model evaluation indicators

In the model evaluation phase, this study selected root mean square error (RMSE), mean absolute error (MAE), sum of squared errors (SSE), normalized root mean square error (nMSE), normalized mean absolute error (nMAE), and coefficient of determination (R²) as evaluation metrics to assess the model’s predictive performance from multiple dimensions, including absolute error, relative error, and goodness of fit. Among these, RMSE, MAE, SSE, nRMSE, and nMAE are error-related metrics, where smaller values indicate higher prediction accuracy. R², as a goodness-of-fit metric, indicates stronger explanatory power with larger values. Specifically noted, there are special cases where the actual value of PV power data is zero (such as zero output during nighttime or extreme rainy weather). This can lead to invalid calculations or infinite values for the Mean Absolute Percentage Error (MAPE). Therefore, this study did not include this indicator in the evaluation system.

All error metrics (RMSE, MAE in kW; SSE in kW^2^) are reported with units, while normalized metrics (nRMSE, nMAE) and R^2^ are dimensionless quantities bounded between 0 and 1.

**Table 2:** Performance of PV power forecasting models on testing set.

Model	RMSE	MAE	SSE	nRMSE	nMAE	*R*²
GRU	0.554	0.481	5388.6	7.72%	6.71%	0.856
LSTM	0.509	0.390	4558.0	7.10%	5.44%	0.878
Transformer	0.361	0.212	2282.6	5.00%	3.00%	0.938
DLinear	0.358	0.200	2246.3	5.00%	2.80%	0.939
CEEMDAN-LSTM	0.337	0.153	1994.4	4.70%	2.14%	0.946
**CEEMDAN-DispEn-LSTM**	**0.326**	**0.112**	**1868.9**	**4.54%**	**1.67%**	**0.950**

The bold values are the optimal values among all methods.

#### Performance under the testing dataset

**Fig. 11:**
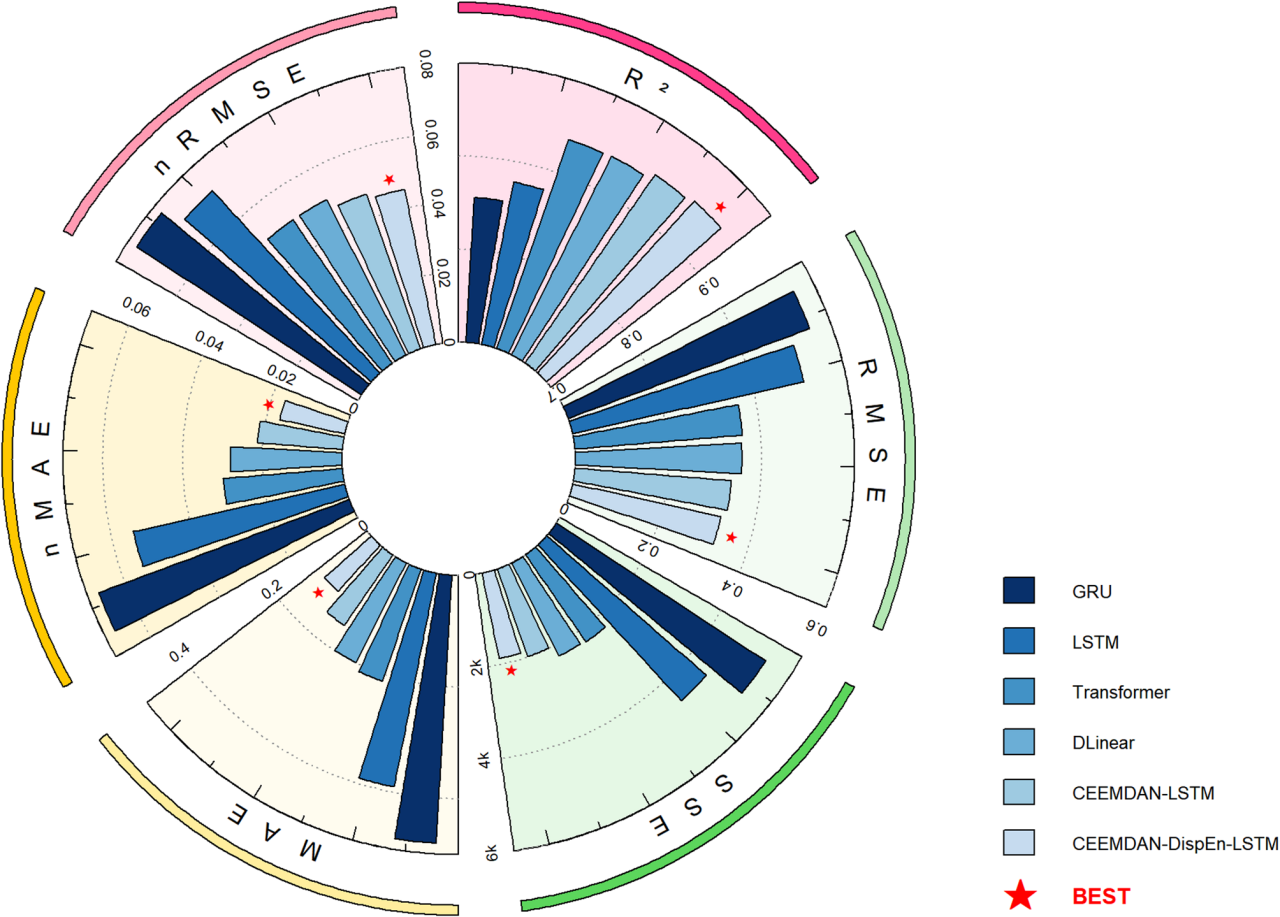
PV power forecasting model model indicator radar comparison chart.

As shown in Table 2; Fig. 11, the CEEMDAN-DispEn-LSTM model exhibits superior performance compared with the comparison models across all evaluation metrics: its RMSE (0.337), MAE (0.189), SSE (1997.08), nRMSE (4.70%), and nMAE (2.63%) are the lowest, while R² (0.947) achieves the highest value. The results indicate that this model significantly improves prediction performance by integrating signal decomposition and feature selection.

**Fig. 12:**
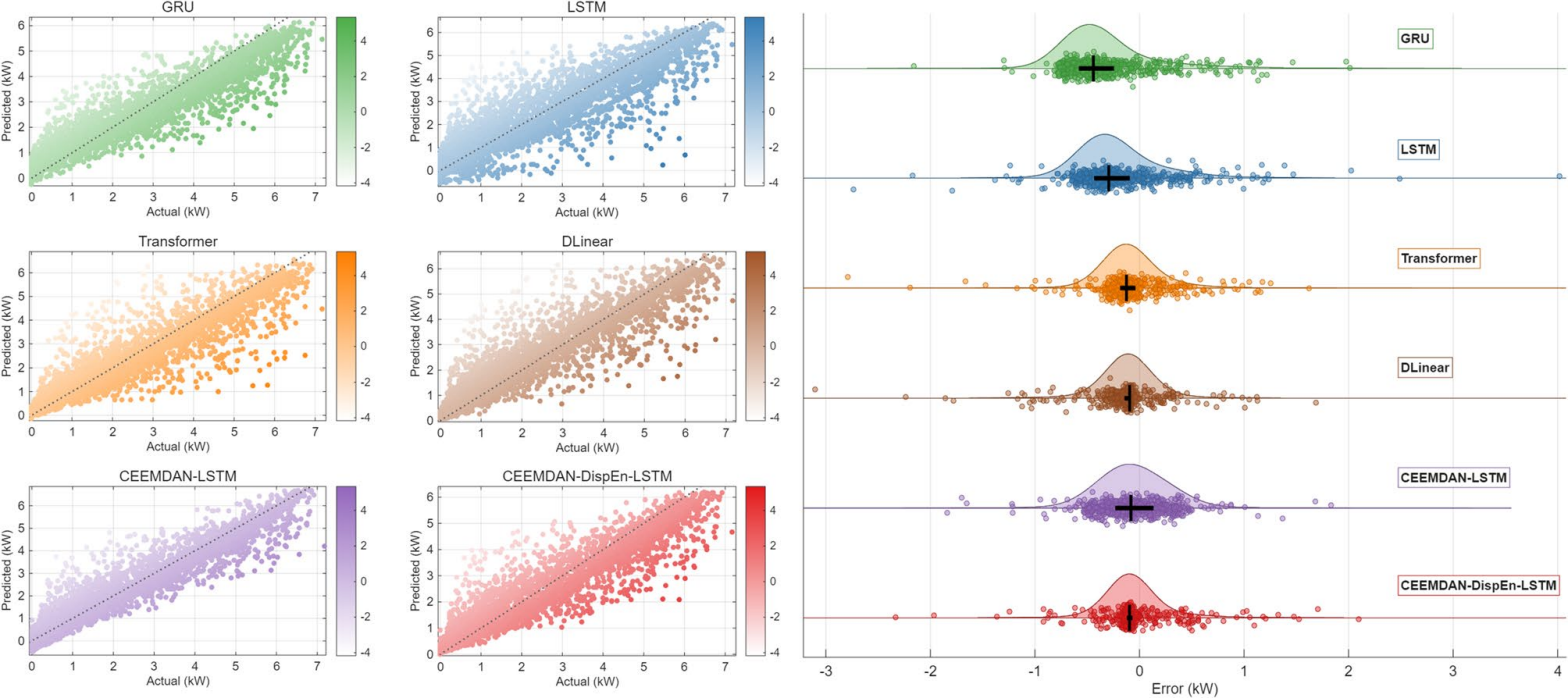
Comparative scatter plots of PV power forecasting models.

In Error Scatter Fig. 12, the predicted points of the CEEMDAN-DispEn-LSTM model are densely distributed near the diagonal line with the lightest shading. Error Cloud Fig. 12 shows that it has the narrowest Interquartile Range (IQR, 0.15–0.20), the most concentrated kernel density curve, and the median line is close to the zero-error line. The visualization results confirm that the prediction accuracy and stability of this model have been improved compared to the comparison models.

**Fig. 13:**
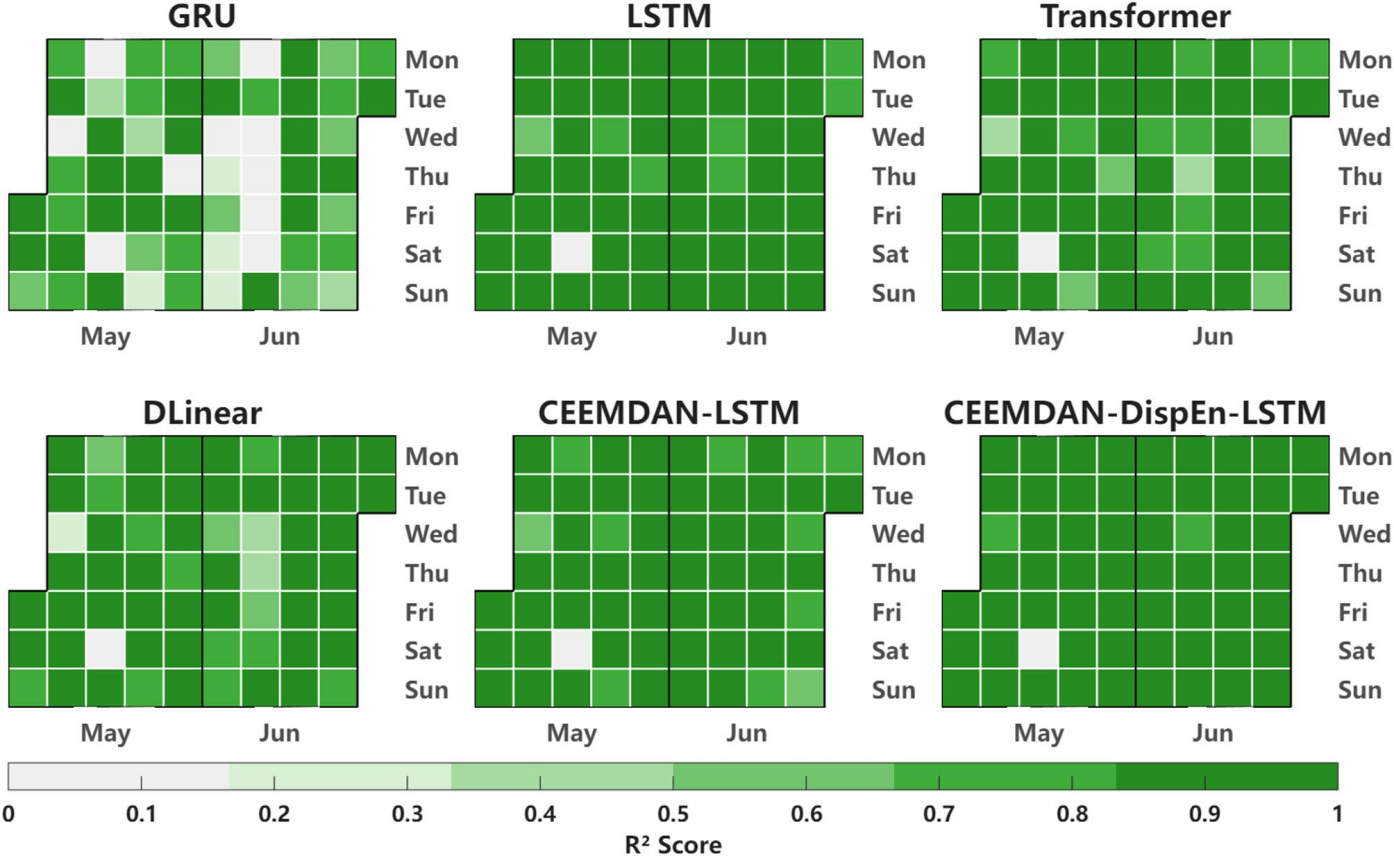
Calendar heatmap of valid prediction days by models.

In the R² calendar heatmap of Fig. 13, the number of dark green blocks (representing R² > 0.9) is the largest (accounting for 85% of the total test days), which is 10%-15% higher than that of the reference models (GRU and LSTM). This further verifies the CEEMDAN-DispEn-LSTM model’s stability and indicates that it can effectively predict most days in the test set.

#### Performance under different weather conditions

**Fig. 14:**
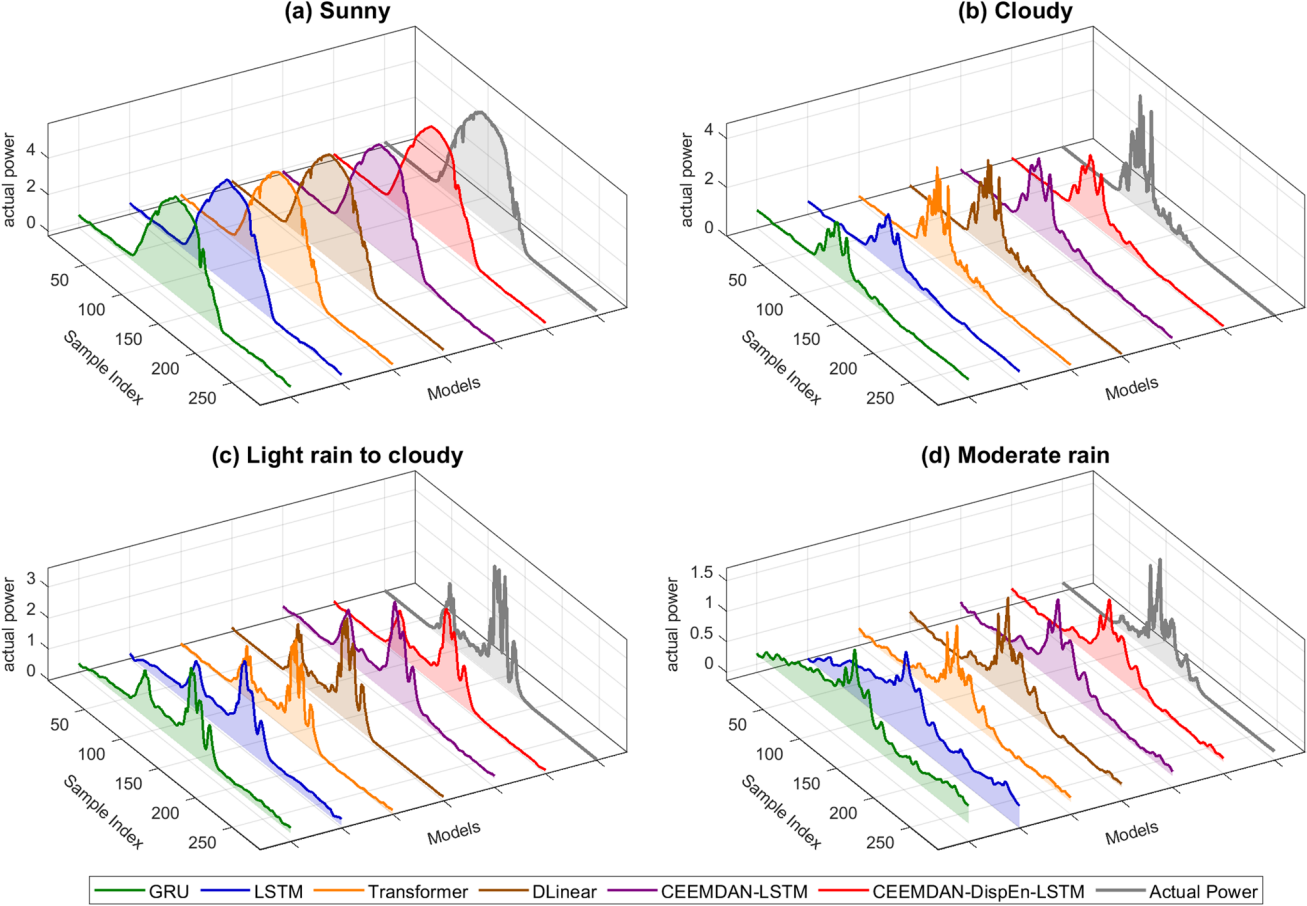
3D curve diagram of prediction results under different weather conditions.

To verify the adaptability of the model, this study selected data from 4 days representing four typical weather scenarios for analysis (Fig. 14):

- Sunny days (stable illumination, periodic power fluctuations): All six models performed well, with the CEEMDAN-DispEn-LSTM model being the best.
- Cloud cover (sudden power changes and significant fluctuations): The reference models performed poorly, the modal decomposition model showed a slight improvement, while the CEEMDAN-DispEn-LSTM model had an obvious advantage.
- Light rain turning to cloudy (intensified power fluctuations): The reference models and the modal decomposition model performed similarly, while the CEEMDAN-DispEn-LSTM model had a significant advantage.
- Moderate rain scenario (low signal-to-noise ratio): The reference models almost failed, the modal decomposition model showed only a slight improvement, while the CEEMDAN-DispEn-LSTM model maintained stable prediction capabilities.

More detailed results are shown in Table 3. Experiments indicate that through the three-tiered collaborative mechanism of “CEEMDAN decomposition → DispEn filtering → LSTM modeling”, the model not only consolidates prediction accuracy in simple scenarios but also breaks through the performance limitations of traditional models in complex scenarios.

**Table 3:** Performance of PV power generation prediction models under different meteorological conditions.

Weather	Model	RMSE	MAE	SSE	nRMSE	nMAE	$\:{\mathbf{R}}^{2}$
Sunny	GRU	0.406	0.367	47.471	0.069	0.062	0.966
	LSTM	0.337	0.288	32.672	0.058	0.049	0.977
	Transformer	0.239	0.161	16.384	0.041	0.027	0.988
	DLinear	0.230	0.143	15.095	0.039	0.024	0.989
	CEEMDAN-LSTM	0.220	0.141	13.832	0.037	0.024	0.987
	**CEEMDAN-DispEn-LSTM**	**0.198**	**0.109**	**11.321**	**0.034**	**0.019**	**0.991**
Cloudy	GRU	0.731	0.698	153.700	0.158	0.151	0.116
	LSTM	0.599	0.551	103.290	0.130	0.120	0.406
	Transformer	0.392	0.291	44.295	0.085	0.063	0.745
	DLinear	0.355	0.200	36.364	0.077	0.043	0.800
	CEEMDAN-LSTM	0.340	0.267	33.300	0.073	0.036	0.780
	**CEEMDAN-DispEn-LSTM**	**0.328**	**0.138**	**30.981**	**0.071**	**0.029**	**0.806**
Light rain turning to cloudy	GRU	0.462	0.425	61.517	0.128	0.117	0.612
	LSTM	0.416	0.345	49.863	0.115	0.095	0.686
	Transformer	0.277	0.160	22.141	0.077	0.044	0.860
	DLinear	0.293	0.175	24.795	0.081	0.048	0.844
	CEEMDAN-LSTM	0.274	0.145	21.676	0.076	0.040	0.863
	**CEEMDAN-DispEn-LSTM**	**0.264**	**0.115**	**20.088**	**0.073**	**0.031**	**0.873**
Moderate rain	GRU	0.435	0.417	54.520	0.262	0.251	-1.483
	LSTM	0.274	0.248	21.571	0.165	0.149	0.018
	Transformer	0.153	0.137	6.711	0.092	0.083	0.694
	DLinear	0.173	0.150	8.639	0.105	0.088	0.607
	CEEMDAN-LSTM	0.117	0.080	3.904	0.070	0.048	0.822
	**CEEMDAN-DispEn-LSTM**	**0.100**	**0.050**	**2.879**	**0.070**	**0.030**	**0.869**

The bold values are the optimal values among all methods.

### Anomaly detection of typical Non-Weather factors

PV power generation systems may encounter various abnormal conditions during operation. Line failure, partial shading, PID (Potential Induced Degradation) effect, and PV module soiling are typical types of faults^42^. These faults not only significantly reduce power generation capacity but may also pose safety hazards. Among them, line failure can cause abnormalities in data collection, leading to data loss or inaccurate values; partial shading typically reduces power generation by approximately 25%; the PID effect results in about 49% power degradation; and PV module soiling, which is often caused by inadequate maintenance, may reduce power generation to nearly zero. This paper focuses on three types of equipment failure anomalies: line failure, partial shading, and PID effect, and does not address the issue of PV module soiling for the time being.

To evaluate the CEEMDAN-DispEn-LSTM model’s capability in detecting equipment fault anomalies, this study selected data from October 12th. For each fault category, ten anomalous data points were injected at 7:00, 12:00, and 17:00 on that day:

- Line Failure: manifested as continuous zero value.
- Partial Shading: manifested as a power decrease of approximately 25%.
- PID Effect: manifested as a power decrease of approximately 40 ~ 49%^43^.

Based on the high-precision prediction values obtained using CEEMDAN-DispEn-LSTM, anomaly detection is performed using the double deviation method. The detection results are shown in Fig. 16, which compares the power changes under different anomaly conditions in a PV power generation system.

**Table 4:** Detection results of non-weather-related anomalies in daily PV operation.

Anomaly Type	Artificially Induced Anomalies	Detected Anomalies	Detection Rate
Line Failure	30	30	100.0%
Partial Shading	30	19	63.33%
PID Effect	30	27	90.00%

**Fig. 15:**
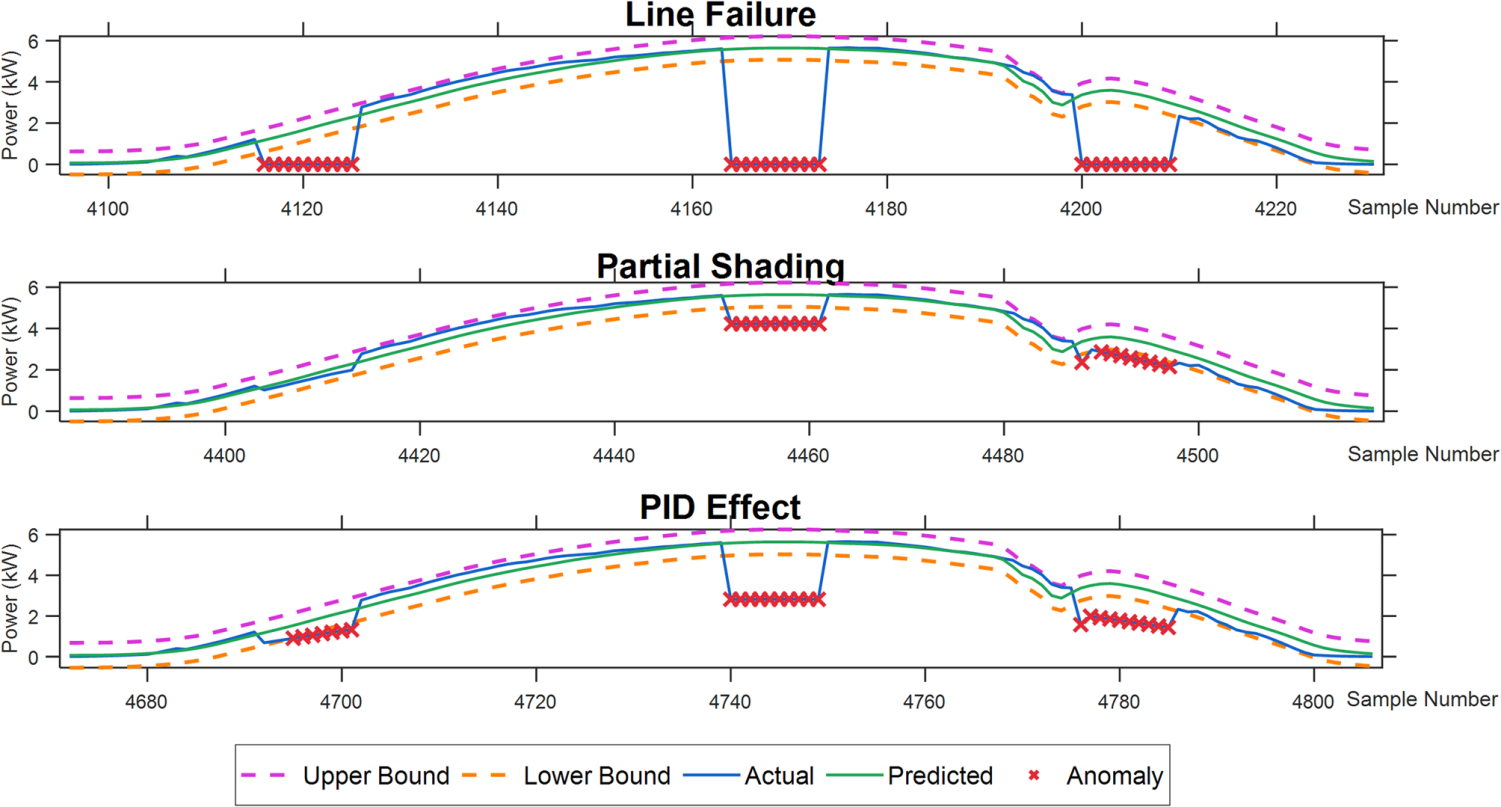
Anomaly detection effectiveness diagram under different abnormal conditions.

Table 4; Fig. 15 demonstrate that the average detection rate reached 84.44%, reflecting the system’s robust comprehensive anomaly recognition capability: Line fault detection rate reaches 100%—this most conspicuous anomaly, characterized by an instantaneous power drop to zero, is easily identifiable at any time.

Partial shading shows the lowest detection rate (63.33%)—the key reasons for this low value lie in its small power fluctuation amplitude and weak distinguish ability from normal variations. Partial shading typically causes only a 25% power reduction, a far smaller magnitude than line faults (instantaneous zero power) or PID effects (40–49% loss). This translates to tiny absolute power changes under low-irradiance conditions (e.g., sunrise/sunset): if normal power is 1–2 kW, a 25% drop is merely 0.25–0.5 kW, which is easily masked by inherent power randomness (e.g., minor irradiance ripples from thin clouds). Moreover, shading-induced power reduction is gradual, overlapping with the slow normal power change of the sun’s rise/set—this makes it hard for the dual-deviation logic to distinguish shading from normal trends. Although detection effectiveness improved at noon (high irradiance amplifies the shading effect), the overall detection rate dropped to 63.33% due to the high proportion of low-irradiance periods within the artificially set anomaly insertion timeframe.

PID effect achieves a 90% detection rate—as an intermediate case, this confirms the principle that “anomaly magnitude determines detection difficulty”: All anomalies become more detectable during midday high-irradiation periods with amplified power fluctuations, compared to reduced sensitivity at sunrise/sunset.

**Fig. 16:**
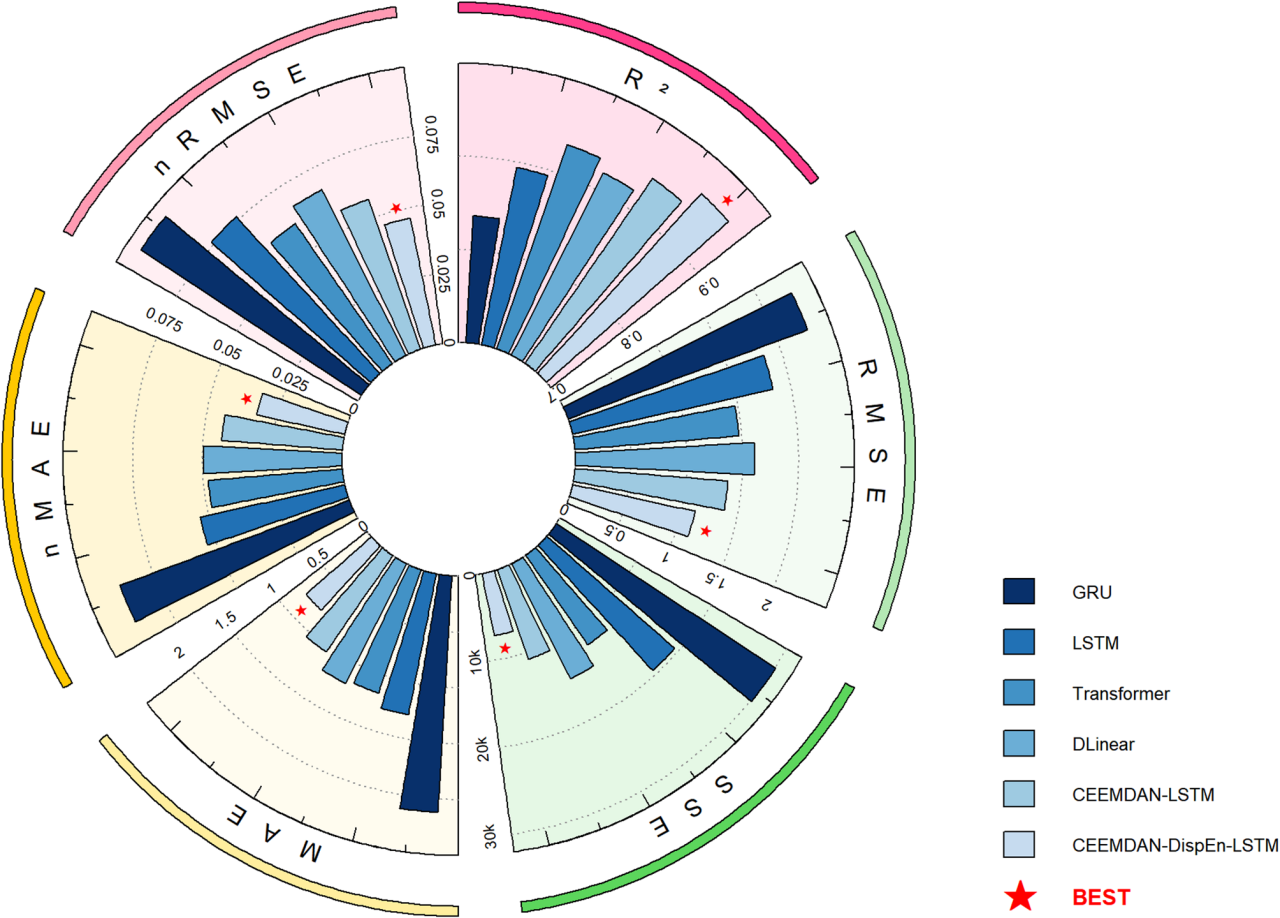
PV power forecasting model indicator radar comparison chart (Dataset #2).

### Validation on CN-SG-REGFC site 8 solar dataset

To further validate the performance of the proposed CEEMDAN-DispEn-LSTM model and other comparative models, additional experiments were conducted using solar power data from Site 8 of the Chinese State Grid Renewable Energy Generation Forecasting Competition^44^ (referred to as CN-SG-REGFC Site 8 Solar Dataset, denoted as Dataset #2 for short). The dataset consists of 15-minute interval measurements, with data from January 1, 2020 to April 30, 2020 selected as the training set and the period from May 1 to June 30, 2020 as the test set. All experimental procedures, including data preprocessing, model configuration, and evaluation metrics, were maintained identical to those applied to the primary dataset to ensure result comparability.

As shown in Table 5; Fig. 16, the CEEMDAN-DispEn-LSTM model demonstrates advantages across most evaluation metrics in the second dataset. Its RMSE (1.334), MAE (0.955), and SSE (10248.0) are 5.3%-8.7% lower than those of the CEEMDAN-LSTM model, and its R² (0.944) is 0.9% points higher. The consistent performance observed across two independent datasets suggests the potential generalizability of the framework for distributed PV systems operating in temperate climates, providing a foundation for future validation across a wider range of environmental conditions.

**Table 5:** Performance of PV power forecasting models on testing set (Dataset #2).

Model	RMSE	MAE	SSE	nRMSE	nMAE	*R*²
GRU	2.287	2.122	30,121	9.47%	8.78%	0.837
LSTM	1.839	1.286	19,481	7.61%	5.32%	0.894
Transformer	1.473	1.166	12,501	6.10%	4.83%	0.932
DLinear	1.606	1.201	14,852	4.97%	4.97%	0.919
CEEMDAN-LSTM	1.374	1.048	10,866	5.68%	4.34%	0.941
**CEEMDAN-DispEn-LSTM**	**1.120**	**0.781**	**7235.6**	**4.63%**	**3.23%**	**0.961**

The bold values are the optimal values among all methods.

**Fig. 17:**
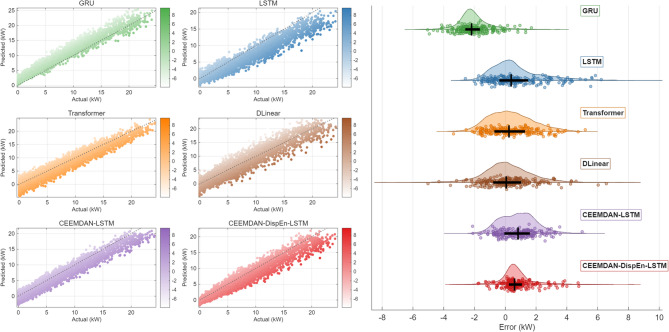
Comparative scatter plots of PV power forecasting models (Dataset #2).

As visualized in Fig. 17, the error distribution of the CEEMDAN-DispEn-LSTM model reveals the following: prediction points cluster densely along the diagonal, indicating high consistency with actual power values. In the error contour plot, although its median line deviates slightly from zero (with an average bias of 0.05 kW), the model exhibits the most concentrated kernel density distribution (with a standard deviation of 0.12 kW) and the narrowest interquartile range (0.18–0.22 kW) among all models. With minimal error dispersion and negligible overall bias, it demonstrates advantages in both prediction accuracy and stability, thereby validating the effectiveness of the results to a certain extent.

**Fig. 18:**
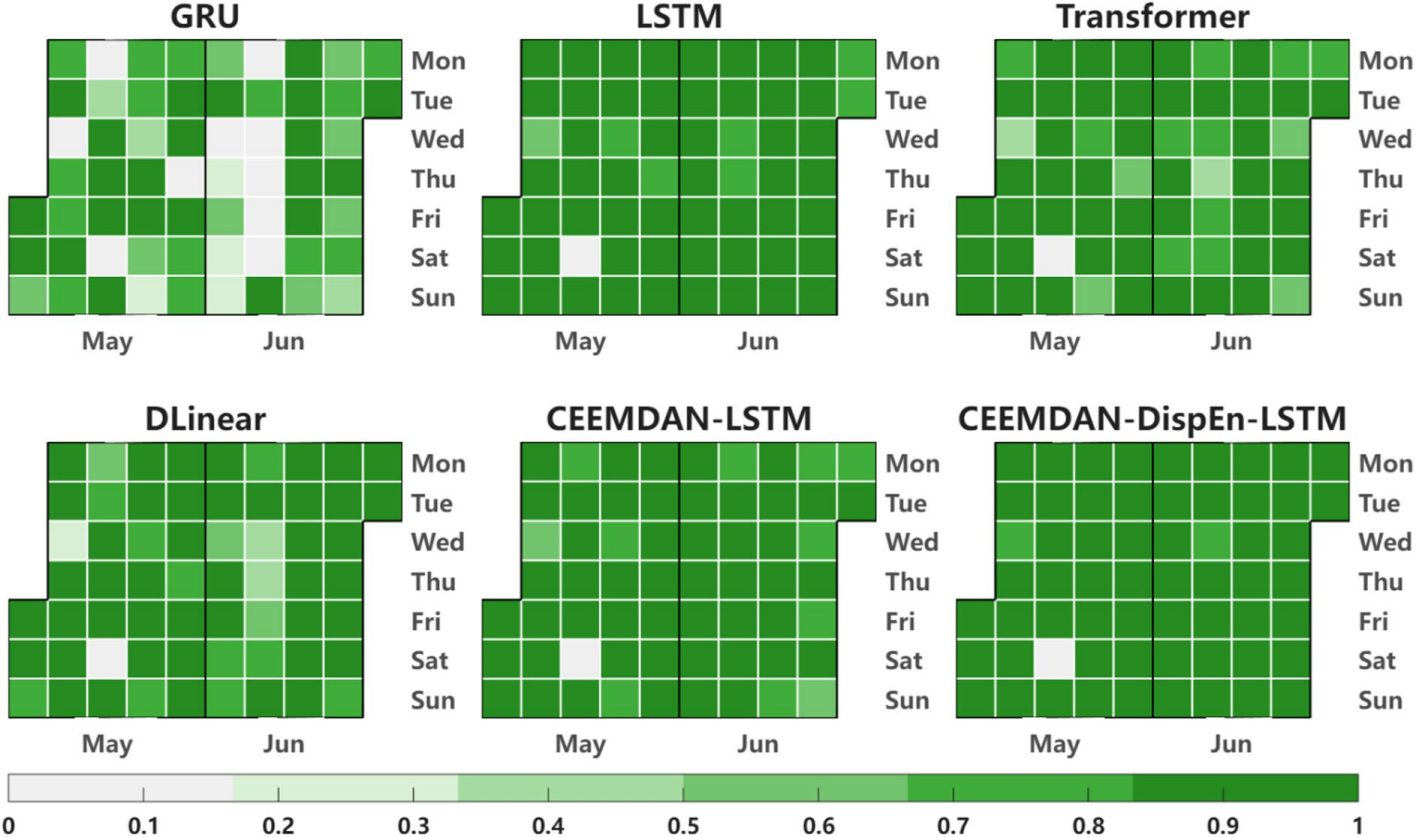
Calendar heatmap of valid prediction days by models (Dataset #2).

Also, in the R² calendar heatmap of Fig. 18, the number of deep green blocks (R² > 0.9) accounts for 80% of the total test days, which is 12%-18% higher than that of the reference models. This further validates the reliability of the dataset validation results.

**Fig. 19:**
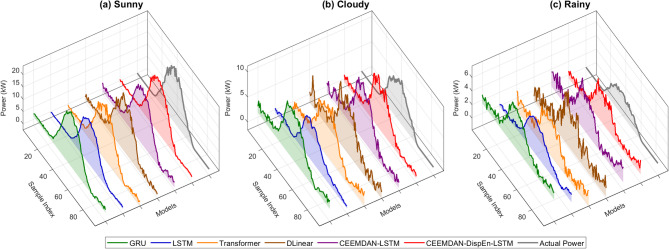
3D curve diagram of prediction results under different weather conditions (Dataset #2).

Dataset validation evaluates a model’s applicability and performance under diverse weather conditions and data distributions. This study analyzed data representing three typical weather scenarios (Fig. 19; Table 6):

- Sunny days (stable insolation): All six models performed well, with the CEEMDAN-DispEn-LSTM model achieving the best results.
- Cloudy days: Reference models performed poorly, whereas the CEEMDAN-DispEn-LSTM model exhibited a distinct advantage.
- Rainy days: Reference models nearly failed; modal decomposition models showed only marginal improvement, while the CEEMDAN-DispEn-LSTM model maintained stable predictive performance.

From the dataset validation results, the CEEMDAN-DispEn-LSTM model achieved optimal performance across multiple metrics under sunny, cloudy, and rainy conditions. This confirms its strong adaptability and predictive capability for PV power datasets covering diverse weather types, thus validating its effectiveness under varied data distributions.

**Table 6:** Performance of PV power generation prediction models under different meteorological conditions (Dataset #2).

Weather	Model	RMSE	MAE	SSE	nRMSE	nMAE	$\:{\mathbf{R}}^{2}$
Sunny	GRU	2.245	2.027	483.840	0.098	0.089	0.919
	LSTM	2.723	1.686	711.540	0.119	0.074	0.881
	Transformer	2.186	1.644	458.600	0.095	0.072	0.923
	DLinear	2.375	1.639	541.630	0.104	0.072	0.910
	CEEMDAN-LSTM	2.246	1.771	484.110	0.098	0.077	0.919
	**CEEMDAN-DispEn-LSTM**	**1.698**	**1.194**	**276.870**	**0.074**	**0.052**	**0.954**
Cloudy	GRU	2.415	2.261	559.880	0.274	0.256	0.355
	LSTM	0.867	0.694	72.213	0.098	0.079	0.917
	Transformer	0.985	0.798	93.085	0.112	0.090	0.893
	DLinear	1.316	0.963	166.130	0.149	0.109	0.809
	CEEMDAN-LSTM	1.187	0.908	135.350	0.135	0.103	0.844
	**CEEMDAN-DispEn-LSTM**	**0.958**	**0.678**	**88.169**	**0.109**	**0.077**	**0.898**
Rainy	GRU	2.739	2.637	720.050	0.778	0.749	-3.41
	LSTM	0.911	0.743	79.737	0.259	0.211	0.512
	Transformer	0.971	0.700	90.587	0.276	0.199	0.445
	DLinear	0.169	0.916	131.250	0.332	0.260	0.196
	CEEMDAN-LSTM	0.840	0.729	69.634	0.232	0.201	0.594
	**CEEMDAN-DispEn-LSTM**	**0.729**	**0.576**	**51.065**	**0.207**	**0.164**	**0.710**

The bold values are the optimal values among all methods.

**Fig. 20:**
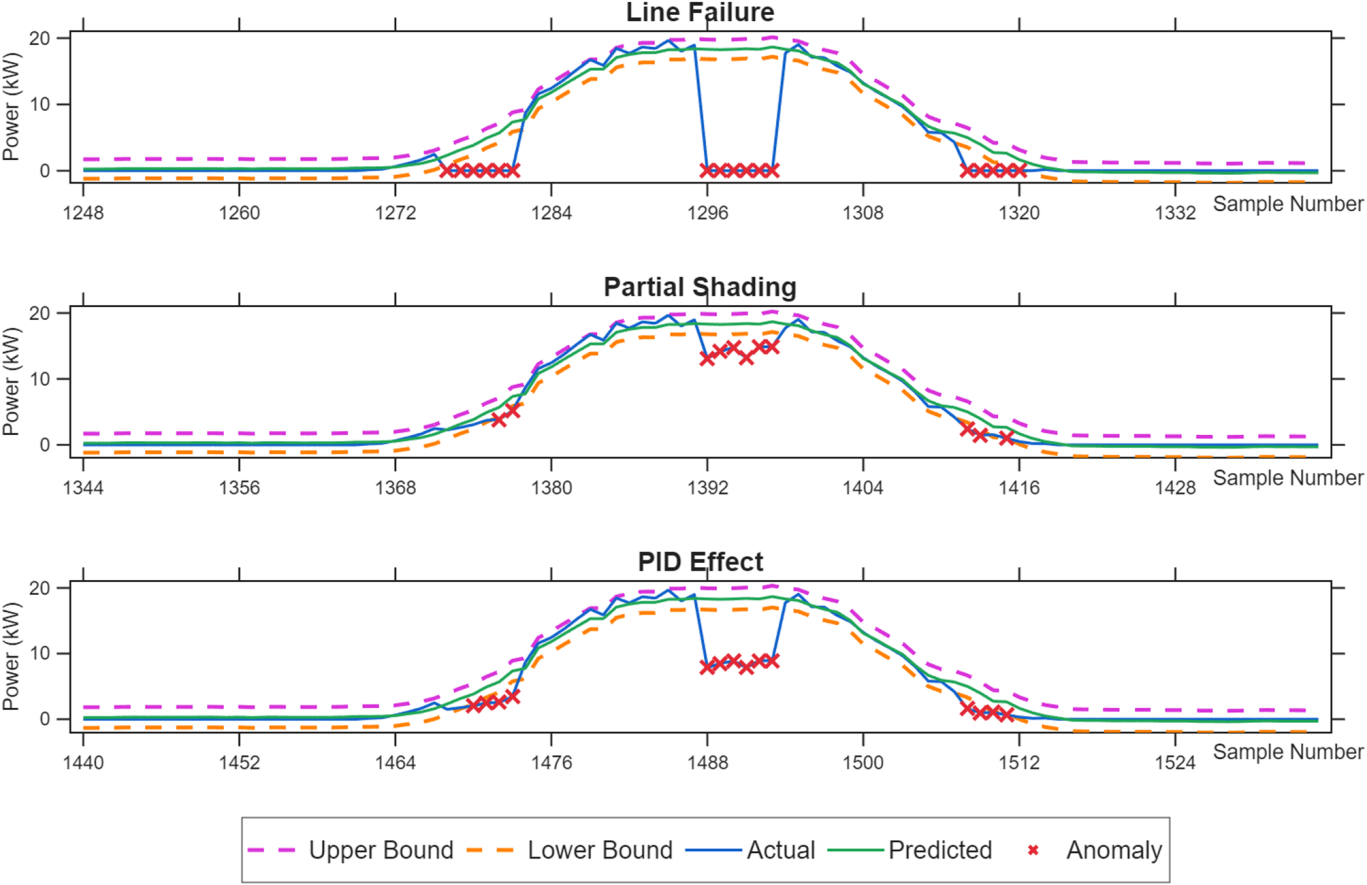
Anomaly detection effectiveness diagram under different abnormal conditions (Dataset #2).

Similarly, to evaluate the system’s ability to detect equipment failures, this study selected data from May 14 and simulated three types of faults, with six anomalous data points injected into each fault category at 7:00, 12:00, and 17:00 on that day. As shown in Table 7; Fig. 20, the line fault detection rate reached 94.44%, demonstrating the best performance; The partial obstruction detection rate was only 61.11%, while the PID effect detection rate reached 77.78%. The overall average detection rate was 77.78%, consistent with expectations. Its detection characteristics align with the results from the first dataset: line fault detection had the highest rate, while partial obstruction detection had the lowest. The causes of this pattern have been explained earlier.

**Table 7:** Detection results of non-weather-related anomalies in daily PV operation (Dataset #2).

Anomaly type	Artificially induced anomalies	Detected anomalies	Detection rate
Line Failure	17	18	94.44%
Partial Shading	11	18	61.11%
PID Effect	14	18	77.78%

## Conclusions

This study proposes a hybrid CEEMDAN-DispEn-LSTM model, integrating CEEMDAN’s adaptive multiscale decomposition and DispEn’s complexity quantification to optimize LSTM input features. Experiments on two data sets show the model outperforms comparison models under tested weather scenarios (sunny, cloudy, etc.), maintains stability across meteorological conditions, and yields improved accuracy versus traditional models.​ Furthermore, the framework achieves high average anomaly detection accuracy. Compared with the adopted traditional residual analysis, it demonstrates advantages that preliminarily validate the multi-scale entropy fusion strategy’s effectiveness for enhancing detection. This work provides an improved method for non-stationary PV power forecasting by optimizing the combination of existing signal decomposition, complexity quantification and deep learning technologies, and offers relevant reference for the application of such hybrid models in smart grid integration.

### Scope of application

The proposed CEEMDAN-DispEn-LSTM hybrid framework is primarily applicable to ultra-short-term power forecasting and real-time anomaly detection scenarios in photovoltaic power generation systems. It effectively addresses power forecasting challenges across PV systems of varying scales. Validation across two independent datasets demonstrates optimal performance under temperate climatic conditions, particularly for PV output forecasting based on inverter sensor data exhibiting pronounced diurnal periodicity.

It should be noted that while the rolling-window CEEMDAN decomposition strategy effectively prevents data leakage, its computational complexity imposes limitations on real-time edge device applications. This primarily manifests as high memory requirements for storing historical window data and substantial computational time needed for decomposition. Additionally, we observe room for improvement in the model’s sensitivity to detecting subtle anomalies, particularly in identifying minor power variations such as partial shading. Under low irradiance conditions, distinguishing abnormal signals from normal fluctuations remains challenging, and the model’s capability for early detection of gradual faults is relatively limited.

#### Limitations and future directions

Geographical and Climatic Generalization: The model’s performance is validated primarily for temperate climates; its efficacy in desert, tropical, or extreme weather regions remains unverified.

Computational Complexity: The practical deployment overhead, particularly from the rolling-window CEEMDAN decomposition, is acknowledged as a constraint for real-time edge applications.

Anomaly Detection Sensitivity: The lower detection rate for partial shading is analyzed, attributing it to the subtlety of the fault’s signature compared to normal weather fluctuations.

Static Model Parameters: The reliance on pre-determined hyperparameters via grid search is noted as a limitation for long-term adaptability against system aging and seasonal shifts.

To address the afore-mentioned limitations, targeted and actionable future research directions are proposed as follows:​.

- For computational complexity constraints: Explore lightweight decomposition algorithms (e.g., optimized CEEMDAN variants or wavelet packet decomposition with reduced computational steps) to minimize processing overhead while preserving decomposition accuracy.​.
- For static parameter limitations: Implement online learning mechanisms to enable dynamic hyperparameter optimization, allowing the model to adapt to long-term system changes and seasonal variations in real time.​.
- For geographical generalization gaps: Integrate high-resolution satellite remote sensing data with ground-based meteorological observations to construct a cross-climate training dataset, thereby developing a more generalized model framework applicable to diverse climatic regions.​.
- For low partial shading detection sensitivity: Incorporate attention mechanisms to enhance the model’s ability to capture subtle fault-related signal variations; alternatively, fuse computer vision data (e.g., drone inspection images of PV arrays) with power signal data to achieve multi-modal fault recognition, improving precision detection for partial shading.

## Data Availability

The PV system dataset used in this study originates from DTU SYSLAB Laboratory and was originally published in Data in Brief article^33^. The complete dataset (SOLETE) is available under the terms spec-ified in the original publication. Processed data subsets generated for this analysis are available from the corresponding author upon reasonable request.The second dataset originates from the China State Grid Renewable Energy Generation Forecasting Competition, as published in Scientific Data in 2022^44^ . For consistency, we used the author’s pre-processed dataset.
